# Engineered species-selective ion-exchange in tuneable dual-phase zeolite composites†

**DOI:** 10.1039/d4sc02664k

**Published:** 2024-08-01

**Authors:** James L. A. Reed, Andrew James, Thomas Carey, Neelam Fitzgerald, Simon Kellet, Antony Nearchou, Adele L. Farrelly, Harrison A. H. Fell, Phoebe K. Allan, Joseph A. Hriljac

**Affiliations:** a School of Chemistry, University of Birmingham Edgbaston Birmingham B15 2TT UK p.allan@bham.ac.uk j.a.hriljac@bham.ac.uk; b Diamond Light Source Ltd, Harwell Science and Innovation Campus Didcot Oxfordshire OX11 0DE UK; c National Nuclear Laboratory, Springfields Salwick Preston PR4 0XJ UK; d Sellafield Ltd, Sellafield Seascale Cumbria CA20 1PG UK

## Abstract

Controllable sorption selectivity in zeolites is crucial for their application in catalysis, gas separation and ion-exchange. Whilst existing approaches to achieving sorption selectivity with natural zeolites typically rely on screening for specific geological deposits, here we develop partial interzeolite transformation as a straightforward and highly tuneable method to achieve sorption selectivity *via* forming dual-phase composites with simultaneous control of both phase-ratio and morphology. The dual-cation (strontium and caesium) exchange properties of a series of granular mordenite/zeolite P composites formed from a parent natural mordenite material are demonstrated in complex, industrially relevant multi-ion environments pertinent to nuclear waste management. The relative uptake of caesium and strontium is controlled *via* the extent of transformation: composites exhibit significantly increased ion-exchange affinity for strontium compared to both the parent mordenite and physical mixtures of mordenite/zeolite P phases with similar phase ratios. The composite with a 40 : 60 mordenite : zeolite P ratio composite achieves higher uptake rates than the natural clinoptilolite material currently used to decontaminate nuclear waste streams at the Sellafield site, UK. *In situ* X-ray image-guided diffraction experiments during caesium exchange demonstrate that the mordenite core retains rapid caesium uptake likely responsible for the unique ion-exchange chemistry achievable through the partial inter-zeolite transformation. These results offer a straightforward and controllable route to optimised zeolite functionality and a strategy to engineer composites from low-grade natural sources at low cost and with formulation advantages for industrial deployment.

## Abstract 2

The partial interzeolite transformation of a natural mordenite presents an effective method to diversify the structure of natural zeolites and enhance their ion-exchange properties.

## Graphical abstract

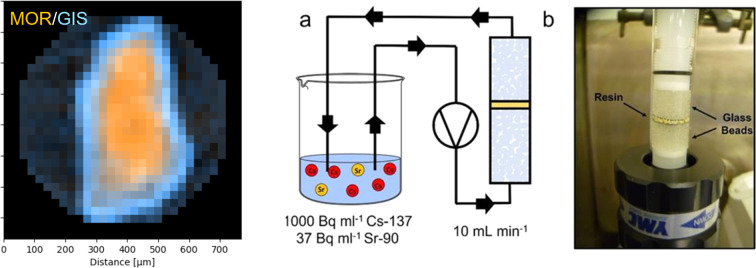

## Introduction

Aluminosilicate zeolites are three-dimensional architectures, comprised of corner-linked silicate and aluminate tetrahedra which form well-defined channels containing exchangeable, charge-balancing cations.^1^ As the tetrahedra can be arranged in many ways there are a variety of different topological structures with a multitude of channel sizes and framework charge densities, giving great versatility to zeolite structure and chemistry.^1^ This versatility, combined with the low cost and high thermal-, chemical- and mechanical-stability of zeolites, has resulted in the extensive employment of zeolites in industrial processes, including catalysis,^2,3^ gas separation^3,4^ and ion-exchange.^3^

Ion-exchange, where extra-framework cations within the structure exchange with others in solution, is integral to applications including the removal of ammonia,^5^ heavy metals^5^ and radionuclides from effluent streams.^6^ Radionuclide uptake from aqueous waste streams is a vital part of routine global nuclear industry operations, where porous materials including zeolites,^6^ hexacyanoferrates,^7,8^ silicotitanates^9,10^ and sodium titanates^8,11^ have been utilised. Abatement of radionuclides is also essential for remediating accidental environmental releases at tragedies such as Three Mile Island (1979),^12^ Chernobyl (1986)^13^ and Fukushima (2011).^14,15^ Cs-137 and Sr-90 are commonly targeted species: they are highly soluble and, combined, account for 99% of medium-lived radioactivity in spent U-235 nuclear fuel.^14,16^ Concentrations of these radionuclides are often very low with comparatively vast quantities of competitive cations (*i.e.* non-radioactive cations which can sit in the structure in place of the cation of interest) present in solution.^14,17^ For example, a waste stream at Sellafield, UK, contained 267 000 Na ions for every Cs-137 nuclide and 59 000 divalent species (Ca and Mg) were in solution for each Sr-90 cation.^17^ Removal *via* ion-exchange must, therefore, be incredibly selective – this is determined by aspects of the zeolite structural chemistry including Si/Al ratio, channel size and shape, in addition to the number and nature of extra-framework cations. When multiple ions need to be removed, the competing chemistry of ions of interest can make achieving this through a single dual-uptake material extremely challenging. This problem has limited the deployment of dual-uptake systems; the majority of materials target a single species, resulting in the requirement to source two materials and adding engineering complexity.^14^ In zeolitic systems, the presence of eight membered rings (8 MR) are the primary selection criteria for high caesium uptake,^18^ but the selectivity of caesium *versus* other univalent competing cations (Na and K) is also important and is promoted by higher Si/Al ratios.^18^ In contrast, for strontium, lower Si/Al ratios are critical in order to have Al–Al pairs present to bind the divalent strontium species.^18^

Ion-exchange must also take place at a suitable rate for column immobilisation. In addition to aspects of the zeolite framework itself, the morphology of particles and monoliths are factors which impact the kinetics of the ion-exchange, as well as the ease of deployment in industrial column systems. Ideally, both the zeolite structural chemistry and morphology would be simultaneously closely controlled and optimised for the composition of the effluent and the deployment system.

Zeolites are typically sourced one of two ways: hydrothermal synthesis from a basic colloidal gel or mining from natural deposits.^19^ Synthetic zeolites have high levels of phase-purity, but are expensive to synthesise and, as they crystallise as micron-scaled particles, often require complex post-synthetic pelleting prior to deployment in industrial systems.^20^ Natural analogues have some advantages which make them attractive for industrial ion-exchange – they are cheaper to source^21^ and easier to obtain as larger, industrially-relevant granules. However, of the over 240 currently known zeolite frameworks,^22^ only around 40 have been found naturally, with only a fraction of these available in significant quantities, making the range of achievable chemistry relatively limited.^23^ Natural deposits also commonly require activation by chemical treatment and often show geological variance, which can impede characterisation and result in disparities in performance which are difficult to rationalise.^6^ A striking example of this is the clinoptilolite (HEU topology) sourced from Mud Hills, California, which has been used as a dual ion-exchanger to remediate 100s m^3^ of Cs-137- and Sr-90-contaminated wastewater per day at the Site Ion-Exchange Effluent Plant (SIXEP) at Sellafield, UK.^6,17^ Like other clinoptilolites, Mud Hills is highly effective for removing Cs-137, but this analogue is unusual in that it outperforms other, seemingly isostructural materials, for Sr-90 uptake. While this poorly understood behaviour has been of great benefit to the nuclear industry,^6,17^ the supply of Mud Hills clinoptilolite is limited, with current stocks forecast to deplete in the 2030s. Further, its performance cannot be tuned to alternative or future feed-stream compositions.

A less common sourcing method is through transformation of another source of silicon and aluminium, such as such as kaolinitic rock,^24^ coal fly ash^25^ or another zeolite. These inter-zeolite transformations take place hydrothermally in alkaline conditions in the presence of a structure directing agent (SDA), commonly a tetra-substituted ammonium species^26–28^ or a metal cation. Sodium is most widely reported to fulfil this role,^29–36^ although transformations utilising other alkali,^28,30,32^ and alkaline earth,^32,37,38^ metals are possible. Metal hydroxides are often used as the sole reagent by providing both an SDA and alkaline conditions. A growing body of work has probed the atomic-scale mechanism of transformation in synthetic (powdered) zeolites.^26,39,40^ The first step is dissolution, whereby the parent zeolite is depolymerised *via* hydrolysis.^41^ Both the solution pH and temperature can control the rate of dissolution, which is routinely considered the rate-determining step. Once the solution concentration of dissolved species reaches the supersaturation threshold, nucleation ensues. Currently, there is no true consensus on the size of the species participating in this nucleation process, although recent work,^42,43^ combined with oligomer size and geometry general reactivity considerations,^44,45^ suggest that smaller ones (small rings and acyclic units) are more likely to be involved. Finally, autocatalytic growth of the structure proceeds until the precursors are sufficiently consumed and there is no supersaturation.^39^ The majority of previous work has focused on complete transformation of the zeolite to a new crystalline or non-crystalline phase.^39^ Whilst the atomic-scale premises of interzeolite transformation should be applicable when applied to the transformation of natural zeolites, natural zeolites are generally used as granules, meaning that both chemical (atomic-scale) and morphological (microscale) processes contribute to the functionality of the material. However, the formation and properties of dual-phase materials from partial interzeolite transformation and the impact upon particle morphology have remained unexplored. Here, we demonstrate that the partial interzeolite transformation of a natural zeolite (mordenite) is a highly controllable method to engineer zeolite composites with tuneable ratios of two phases with complementary ion-exchange chemistry. Concurrent morphological control is obtained *via* preservation of the parent granule morphology.

We obtain a range of two-phase zeolite composite dual-ion-exchangers from a single low-grade natural mordenite source. Mordenite (MOR topology), a zeolite of high geological abundance, displays excellent affinity for caesium^18^ but suffers from poor strontium-uptake, meaning it is excluded as a potential dual-ion-exchanger in the nuclear industry. The inter-zeolite transformation *via* a hydrothermal NaOH treatment^35,46,47^ is known to generate a more aluminous zeolite P (GIS topology) phase, which shows increased cation capacity and enhanced Sr^2+^ uptake.^35,46,47^ This originates from the additional charge-balance required by the additional framework aluminium, stronger interactions between the more negatively charged framework and charge-dense, divalent Sr^2+^ cations and the presence of more aluminium pairs within the framework. Previous studies also highlight a small decrease in Cs^+^ uptake, although rate of uptake for both species increased.^46^ While mordenite structures are known to transform to zeolite P, previous studies have focused on using high concentration NaOH solutions (>2 M)^35,47^ to ensure complete transformation into the GIS structure.^46^ Here we show that partial interzeolite transformation allows the relative uptake of strontium and caesium ions of granular materials to be closely and predictably controlled. This offers a straightforward strategy to engineer optimised properties from low-grade natural sources at low cost, and with formulation advantages for industrial deployment.

## Methods

### Materials

A mordenite material was sourced from Java, Indonesia.

### Inter-zeolite transformation of MOR powder

The raw mordenite material was crushed and sieved to a particle size <75 μm, ensuring homogeneity. A 0.667 g portion of the zeolite was added to a 60 mL polypropylene bottle charged with 23 mL NaOH solution (Alfa Aesar 98%, 0.2–0.7 M) and heated at 100 °C for 24 h. After cooling, the solid material was washed extensively with distilled water and dried at 60 °C for 16 h. Powder X-ray diffraction patterns were subsequently measured on a Bruker D8 diffractometer (Cu source, K_α1_ and K_α2_ radiation, *λ* = 1.5406 Å and 1.5444 Å respectively). The Si/Al atomic ratio was determined by Wavelength Dispersive X-ray Fluorescence (WDXRF) measurements conducted on a Bruker S8 Tiger, using pelletised material (20% SpectroBlend® 44 μm binder, pressurised for 3 minutes at 8 tonnes).

### Activation of mordenite

Raw, hand-ground material (4 g, <75 μm) was washed with distilled water and dried at 60 °C prior to addition to a 40 mL 1 M NaCl solution (Fisher, 98%). After 24 h of agitation, the remaining solid was washed with distilled water and left to dry at 60 °C for 16 h.

### Batch ion-exchange testing

Ion-exchange testing solutions of the following compositions were made up through dilution of inductively coupled plasma (ICP) standards (Fisher): 10 ppm Cs, 50 ppm K; 10 ppm Sr, 50 ppm Ca. A 0.03 g portion of the relevant material was added to 15 mL of each solution and agitated for 24 h before extraction of supernatant, which was subsequently passed through a 0.22 μm filter, acidified with 2% nitric acid (VWR, ultrapure) and analysed by inductively coupled plasma mass spectrometry (ICP-MS). The percentage uptake was calculated using eqn (1).1
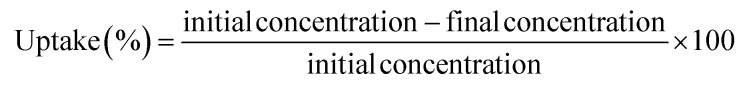


### Adsorption studies

For caesium sorption studies 15 mL of 50–500 ppm Cs solution (CsNO_3_, Alfa Aesar 99.8%) was added to a polypropylene tube charged with 0.018 g zeolite material prior to agitation for 30 days at ambient temperature. Subsequent supernate was extracted, passed through a 0.22 μm filter, diluted in 2% nitric acid (VWR, ultrapure) and analysed by ICP-MS. For strontium sorption studies, 15 mL of 10–500 ppm Sr solution (Sr(NO_3_)_2_, Fisher analytical reagent grade) was added to a polypropylene tube charged with 0.018 g zeolite material prior to agitation for 30 days at ambient temperature. Subsequent supernate was extracted, passed through a 0.22 μm filter, diluted in 2% nitric acid (VWR, ultrapure) and analysed by inductively coupled plasma optical emission spectroscopy (ICP-OES). Each experiment was performed in triplicate. For further details on data analysis please refer to the ESI 7.†

### Formation of granular composites

The raw mordenite material was crushed and sieved to a particle size of 400–600 μm. A 0.667 g portion of zeolite was added to a 60 mL polypropylene bottle charged with 23 mL NaOH solution (Alfa Aesar 98%, 0.7–1.2 M) and heated at 100 °C for 24 h. After cooling, the solid material was washed extensively with distilled water and dried at 60 °C for 16 h. Powder diffraction patterns were subsequently measured on a Bruker D8 diffractometer (Cu source). Images of specimens examined on a Hitachi TM4000+ scanning electron microscope (SEM).

### DIAD beamline experiments

X-ray imaging and diffraction were conducted on the Dual Imaging and Diffraction (DIAD) instrument at Diamond Light Source.^48^ The instrument's optical elements were configured to provide a pink imaging beam (low angle, Pt mirrors, 4 mm Al filter^48^) alongside a focused 25 μm × 25 μm monochromatic beam used for diffraction. Monochromatic light was produced by reflection through a double crystal monochromator equipped with Si (111) crystals. During the experiment the wavelength was calibrated at 0.5929 Å by observing points on Debye–Scherrer rings produced by a certified LaB_6_ powder.^49^ Geometric instrument parameters were further refined in DAWN.^50^

#### Instrument detector settings

Imaging data were collected using the PCO EDGE 5.5 system, with diffraction data being captured using a Pilatus 3 × 2 M CdTe detector.^48^ The imaging detector's exposure time was determined based on the time to nearly fill the dynamic range with the flat field, 60 ms per frame. Acquisition time for the diffraction frames depended on the acquisition mode; in single point mode (here referred to as point-and-shoot) an acquisition time of 30 s was used, when taking diffraction tomography measurements, long scan times necessitated a reduction in the acquisition time to 5 s per frame. The imaging system was positioned at 150 mm away from the sample to maximise the angular coverage of the Debye–Scherrer rings visible to the diffraction detector. The radius of the detector was chosen following similar argumentation to provide a final diffraction detector position of radius = 322 mm, alpha = 26°, beta = 16.75°.^51^

#### Acquisition settings

Individual tomograms consisted of 2500 projections over 180°, with 20 flat and 20 dark frames taken at the start and the end of the tomography acquisition. A long-duration 7-slice diffraction tomography scan was conducted on a composite granule using 91 rotations steps over 360°. For further information regarding calibration, specimen mounting, positioning and alignment and data analysis pipelines please refer to ESI 1.†

#### *In situ* studies of ion-exchange

A single granule of relevant material was mounted on a polyamide rod adhered to the interior of a borosilicate capillary. The granule was subsequently centred on the beam prior to addition of 5000 ppm Cs solution (CsNO_3_ Alfa Aesar 99.8%). This liquor was contained by the capillary throughout the experiment, ensuring constant exposure of the granule. Time-resolved diffraction patterns were repeatedly collected at regions of interest using a 30 seconds exposure time. Integrated intensities of key peaks were calculated using OriginPro.

### Rapid ion-exchange (RIX) testing of granular composites with active nucleotides

**Fig. 1:**
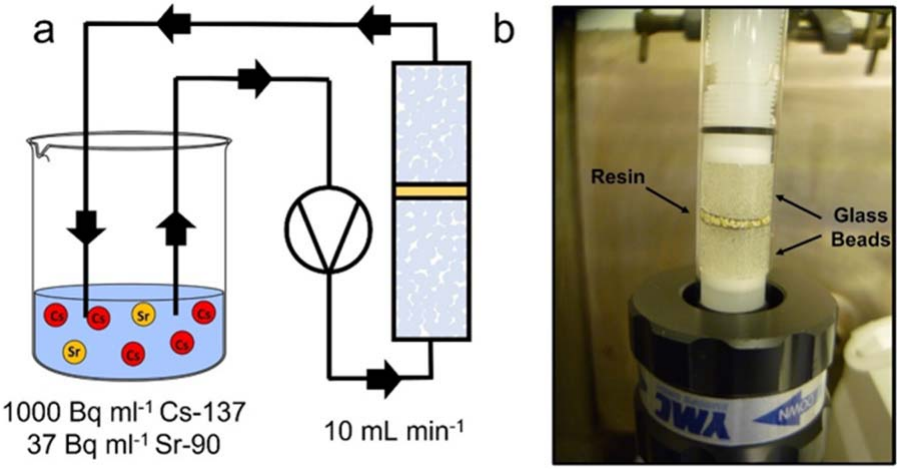
RIX experimental setup. (a) RIX schematic. (b) Photograph of loaded column.

A 1 cm diameter column was loaded with 0.1 g ion-exchange material as slurry, surrounded (top and bottom) by 0.5 g 1 mm glass beads, separated by a stainless steel mesh (Fig. 1b). The loaded column was connected to a peristaltic pump, operating at a flow rate of 10 mL min^−1^. Ultrapure water flowed through the system for specific time intervals in order to accurately determine flow rate for each experiment. A simulant liquor was prepared to replicate the caesium (0.017 ppm) and strontium (0.0005 ppm) concentration expected in the SIXEP feed stream, similar to that reported previously.^6^ An aqueous stock (pH 7–8) was prepared by spiking the solution with Sr-90 (37 Bq mL^−1^) and Cs-137 (1000 Bq mL^−1^) isotopic standards, and adding inactive CsCl to prepare the desired concentration. Active simulant liquor was pumped through the column and recirculated back into the liquor reservoir (Fig. 1a). At *t* = 0, 7, 15, 45, 90, 180 minutes, 1 mL aliquots were withdrawn from the reservoir and stored in glass vials for analysis. This procedure was performed in triplicate for each material. Cs-137 activity was determined by gamma spectroscopy on GMX-20190 and GMX-18190S N type spectrometers. Sr-90 activity was determined *via* Cherenkov counting on a TRI-CARB® Liquid Scintillation Analyser, Model 2700 TR Series. Solution activities were reported as a percentage of starting activity (*t* = 0) and plotted as a function of volume (through column, *V*_c_). A two-parameter exponential decay (eqn (2)) function was fitted to these points using a Levenberg Marquardt iteration algorithm until convergence was reached (Chi-Sqr tolerance value 1 × 10^−9^). This curve then represents all data for a single material.

2 *y* = *y*_0_ +  *A*_1_e_1_^−*x*/*t*^ + *A*_2_e_2_^−*x*/*t*^

This method allows for dynamic flow testing in a very short timeframe, requiring much smaller quantities of both ion-exchange material and solution in comparison to traditional column studies, resulting in reduced waste and costs.

## Results and discussion

### Synthesis of powder composites

**Fig. 2:**
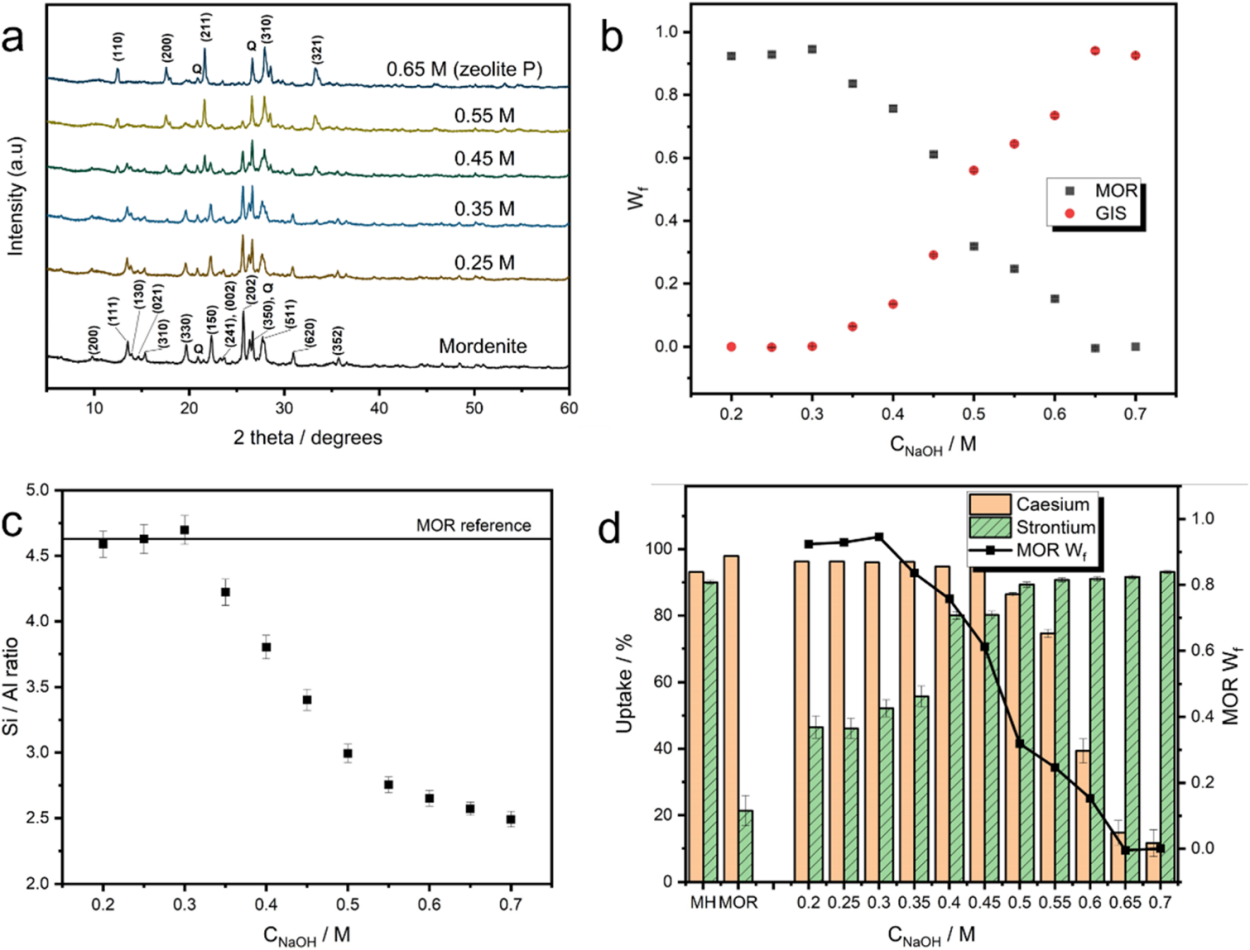
Analysis of zeolite composites. (a) Selected PXRD patterns of zeolite composite materials derived from mordenite (*λ* = 1.5406 Å). NaOH treatment concentrations are labelled. Key MOR and GIS reflections are labelled on the mordenite and 0.65 M patterns respectively, in addition to the reflections for quartz (*Q*). All PXRD patterns are available in the ESI 2.† (b) Weight fraction (*W*_f_) of MOR and GIS phases present in the zeolite composites as a function of NaOH concentration (*C*_NaOH_), as determined from Rietveld refinements (error bars present but smaller than points, refinements assumed presence of MOR, GIS and quartz). (c) Si/Al ratio of materials as a function of NaOH concentration (*C*_NaOH_), as determined by XRF measurements. (d) Batch uptake data for caesium and strontium for the series of composite zeolites, in addition to activated Mud Hills clinoptilolite (MH) and mordenite (MOR). The mordenite weight fraction is also shown for reference.

The partial transformation of mordenite into MOR/GIS composite zeolites was achieved through hydrothermal treatment with NaOH. Eleven materials were generated using eleven different NaOH concentrations between 0.2 M and 0.7 M. Rietveld analysis^52,53^ of powder X-ray diffraction (PXRD) patterns (Fig. 2a, ESI 3†) revealed that the growth of the GIS-type phase was observed above 0.3 M and increased steadily up to approximately 0.65 M, where negligible amounts of the mordenite material remained (Fig. 2b). This demonstrates the transformation can be controlled through choice of the NaOH concentration, allowing for close and predictable control of the phase ratio in the materials. X-ray fluorescence (XRF) spectroscopy highlighted a gradual reduction in bulk Si/Al ratio above 0.3 M NaOH concentration (Fig. 2c). This can be attributed in bulk to the nucleation of a more aluminous structure following parent dissolution which is reported in the MOR/GIS transformation,^47^ with silicon being lost from the system; this is reflected in the Si/Al ratio of ∼2.5 at concentrations above 0.7 M, where the transformation is complete. However, it is also likely that the remaining mordenite in the partially transformed materials is also somewhat desilicated in comparison to the parent structure. The extent of this desilication can be estimated *via* the discrepancy observed between the Si/Al ratio attained by XRF and the Si/Al ratio calculated for a combination of MOR and GIS phases based on relative weight fractions from Rietveld refinement, assuming that the end-member Si/Al ratios of 4.6 and 2.5, respectively, are unchanged throughout (ESI 4†). This analysis revealed an estimated decrease in Si/Al ratio from 4.6 to 3.4 for the mordenite phase during the transformation (up to 0.60 M).

### Batch ion-exchange testing

The dual-ion uptake of composite materials was probed using batch ion-exchange trials. These were performed on the eleven materials with variable MOR/GIS fractions, in addition to an activated (Na-exchanged) sample of parent mordenite and Mud Hills clinoptilolite, which is deployed industrially at the Sellafield site, UK. The uptake of caesium- and strontium-ions were measured separately and in excess potassium and calcium, respectively; although effluent solutions will contain large quantities of other ions, such as sodium, these are the main competing cations for ion-exchange as a result of their chemical similarities.

Uptake data from these experiments is summarised in Fig. 2d; the parent mordenite achieves exceptional (>96%) caesium uptake, which can be attributed to it containing both twelve- and eight-membered ring channels in its structure and its comparatively silicious framework.^18^ The high uptake is retained in composites containing approximately more than 60% of the MOR phase (converted in 0.45 M NaOH). At higher conversion levels, Cs-uptake rapidly decreased, with the fully converted (GIS) material showing Cs uptake of around 10%.

For strontium, the Mud Hills clinoptilolite exhibits excellent affinity (90% removal), consistent with previous literature which demonstrates an unusually high affinity compared to other clinoptilolites.^6,54^ For the partially and fully transformed materials, strontium uptake followed the opposite trend to caesium: the parent mordenite shows poor (∼20%) uptake, due to the high Si/Al ratio meaning there are relatively few Al-pairs suitable for binding divalent strontium. The strontium uptake increases rapidly in materials converted in up to 0.5 M NaOH (approximately 70% conversion, when Cs affinity also remains high) and continues to rise to maximum of 90% when fully-converted to GIS in 0.7 M NaOH. GIS-type zeolites have more aluminous frameworks, favouring the uptake of the more charge dense Sr^2+^, which requires two nearby aluminium tetrahedra to bind.^18,46^ Interestingly, strontium removal improved significantly in 0.20 and 0.25 M treatments when compared to parent mordenite, when no growth of a crystalline GIS-type phase or change to Si/Al ratio was detected. This is possibly due to initial desilication of the mordenite material at the surface (prior to significant crystallisation of zeolite P), or dissolution of amorphous content within the parent material. N_2_ porosimetry (ESI 5†) shows a reduction in surface area in a sample treated for 24 hours with 0.2 M NaOH with the loss of pores between 2 and 5 nm and the growth of pores between 5 and 30 nm, similar to other mild treatments previously reported^55^ and consistent with desilication leading to larger porous areas. Full-width half maximum of reflections in XRD data show insignificant changes (ESI 6†), indicating that the zeolite structure remains unchanged. The ratio of mordenite/zeolite P within the powder composites was shown to tune the relative uptake of the two ions (Fig. 2d). The material formed using a 0.5 M treatment, containing an approximately 2 : 3 weight ratio MOR : GIS in the composite, displays optimal dual-uptake from within these cation matrices (86% Cs uptake, 89% Sr uptake). This performance is comparable to that of Mud Hills clinoptilolite in the same conditions (93% Cs uptake, 90% Sr uptake). A 50 : 50 physical mixture of the starting mordenite (Na-exchanged) and ‘fully converted’ zeolite P (*i.e.* the material transformed in 0.7 M NaOH where no mordenite was observed in the XRD) were also tested in equivalent conditions; 94 and 67% of caesium and strontium were removed, respectively. Based on our data, a 50 : 50 composite material formed through hydrothermal conversion would be expected to remove approximately 90% of caesium and 85% of strontium; this increased strontium (and slight decrease in caesium) uptake is attributed to the before-discussed partial desilication of the parent mordenite which occurs concurrently to the partial inter-zeolite transformation (ESI 4†).

### Adsorption studies of selected materials

The maximum sorption capacities, *q*_max_, for caesium and strontium were determined through analysis of adsorption isotherms (ESI 7†) for four materials in the absence of competing cations: Mud Hills clinoptilolite, parent mordenite, MOR/GIS composite (0.50 M NaOH, MOR : GIS ratio 32 : 56) and the material fully converted zeolite P (0.70 M NaOH). These values are summarised in Table 1, which also includes data from a variety of the most widely industry-used materials for comparison. Information on alternative non-industrially-deployed materials is summarised in ESI 8.† The transformation from the parent mordenite to zeolite P results in an increase in caesium capacity from 167 mg g^−1^ to 206 mg g^−1^; this is likely a result of the increasingly aluminous materials (from both newly-crystalised zeolite P and desilication of parent mordenite), as revealed by XRF analysis. Mimura^46^ reported a slightly higher, but comparable, caesium capacity of 234 mg g^−1^ in synthetic zeolite P. The caesium capacity of the composite (209 mg g^−1^) and fully-converted material compare favourably with that measured for both Mud Hills clinoptilolite and reported values for Ionsiv IE-911.^9^

**Table 1:** Caesium and strontium capacity comparison of selected materials to a host of industrially deployed ion-exchange materials used for abatement of caesium/strontium. Information on non-industrially deployed materials is summarised in ESI 8

Material	Structure type	Industrial deployment	Source	*q*_max(Cs)_/mg g^−1^	*q*_max(Sr)_/mg g^−1^
Mud Hills Clinoptilolite	Zeolite (HEU topology)	Cs, Sr at Sellafield, UK^17^	Natural (California)	203(24)	57(4)
Ionsiv® IE-911	Crystalline silicotitanate (CST)	Cs, (Sr) at Hanford, WA, USA^9^	Synthetic	252(67)^9^	—
CsTreat®	Hexacyanoferrate	Cs at Fortum Loviisa, Finland, JAERI site (Japan), UKAEA (UK), Callaway (USA), Paks (Hungary), Fukushima Daiichi (Japan)^14^	Synthetic	46.5 (ref. 14)	—
SrTreat®	Sodium titanate	Sr, JAERI (Japan), Fukushima Daiichi (Japan)^14^	Synthetic	—	219(13)^14^
Mordenite	Zeolite (MOR topology)	—	Natural (Java)	167(10)	54(16)
MOR/GIS composite (0.50 M NaOH)	Zeolite (MOR/GIS topologies)	—	This work	209(8)	130(22)
Zeolite P (0.70 M NaOH)	Zeolite (GIS topology)	—	This work	206(24)	146(15)

Strontium capacity increases markedly as the transformation proceeds from 54 mg g^−1^ to 156 mg g^−1^ for the fully converted zeolite P: the more aluminous framework will contain more aluminium pairs to which strontium can adhere. This is comparable to work by Mimura,^46^ who reported a capacity of 161 mg g^−1^ for zeolite P (also synthesised from natural zeolites). The strontium capacity of the composite material (130 mg g^−1^) shows over double the strontium capacity of Mud Hills clinoptilolite, and although it remains lower than reported for the strontium-only ion-exchanger, SrTreat©, the simultaneous high capacity and selectivity for Cs adds appeal as a dual-uptake material.

Taken together with the batch ion-exchange experiments performed in the presence of competitive cations, the data show that the loss of Cs uptake observed in batch ion-exchanges for the transformed materials is likely due to the decrease in selectivity for Cs over K, as the Si/Al ratio reduces during the transformation, rather than a decrease in the gravimetric capacity: in batch ion-exchange, only 2–3% of the gravimetric capacity of the materials is utilised if all caesium is taken up from the solution. This is consistent with previous work by Kwon^18^ who found that univalent exchanges for less charge dense cations are promoted by high Si/Al ratios, and become less selective as silicon content reduces.

In the case of strontium, Mud Hills clinoptilolite and the parent mordenite have similar gravimetric uptake capacities, and yet the performance in batch ion-exchange studies is vastly different despite only a low proportion (between 3 and 13.5%) of the full capacity being required for full uptake in batch ion-exchanges (due to the enhanced selectivity of strontium over calcium). For the partially transformed materials, the increased uptake in the composites must originate, at least in part, from the large increase in strontium capacity observed as the zeolite P phase forms, although the selectivity for Sr over Ca may also be modified by the transformation.

### Formation of granular composites

**Fig. 3:**
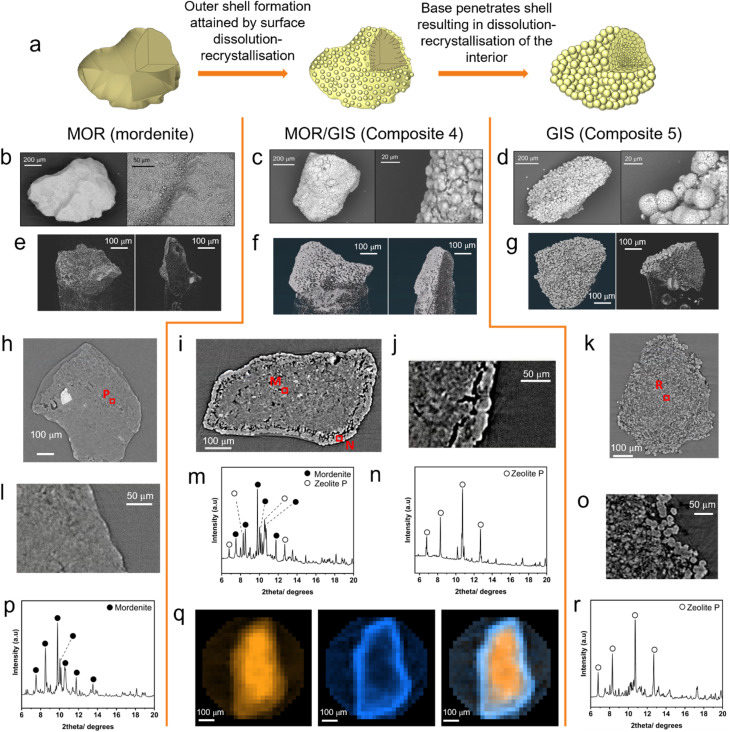
Mechanism of transformation derived from imaging and local X-ray diffraction patterns. (a) Proposed macroscale-mechanism of interzeolite transformation. (b) SEM images of mordenite granule. (c) SEM images of composite (Composite 4) granule. (d) SEM images of GIS (Composite 5) granule. (e) Three-dimensionally reconstructed tomography images of a mordenite particle (left) and a dissected mordenite particle (right). (f) Three-dimensionally reconstructed tomography images of a Composite 4 particle (left) and a dissected Composite 4 particle (right). (g) Three-dimensionally reconstructed tomography images of a GIS Composite 5 particle (left) and a dissected Composite 5 particle (right). (h) Tomography cross-section of mordenite granule and diffraction beam trajectories for data presented in part (p). (i) Tomography cross-section of Composite 4 granule and diffraction beam trajectories for data presented in parts (m) and (n). (j) Zoomed-in tomography cross-section of Composite 4 granule. (k) Tomography cross-section of Composite 5 and diffraction beam trajectory for data presented in part (r). (l) Zoomed-in tomography cross-section of mordenite granule. (m) Processed diffraction data collected at point M (*λ* = 0.5965 Å). Key reflections form the MOR and GIS phase are labelled. Further information about impurity phases can be found in ESI 8.† (n) Processed diffraction data collected at point N (*λ* = 0.5965 Å). Key reflections form GIS phase are labelled. Further information about impurity phases can be found in ESI 8.† (o) Zoomed-in tomography cross-section of Composite 5 granule. (p) Processed diffraction data collected at point P (*λ* = 0.5965 Å). Key reflections attributed to mordenite are labelled. (q) Spatially-resolved diffraction tomography exhibiting phase distribution of MOR and GIS phases, in a granule of Composite 4. Left: distribution of MOR framework determined by integration of (620) Bragg reflection peak. Centre: distribution of GIS framework determined by integration of (200) Bragg reflection peak. Right: overlay of MOR and GIS phase maps. (r) Processed diffraction data collected at point R (*λ* = 0.5965 Å). Key reflections attributed to the GIS phase are labelled; further information about impurity phases can be found in ESI 11.†

Having the material in granular form is often essential for industrial ion-exchange applications, which routinely operate in dynamic flow systems where effluent is pumped through a fixed bed of ion-exchange material.^8,9,11,17,56^ Granular composites, analogous to powder varieties previously discussed, were attained *via* pre-sieving of the mordenite precursor to a particle size of 400–600 μm, followed by NaOH treatment. Higher NaOH concentrations were required to form granular composites with the same phase ratios as in powder transformations (Table 2), presumably due to decreased surface area, resulting in reduced kinetics of dissolution. SEM images suggest that the granule size is retained throughout the transformation process (Fig. 3b–d). These images show the formation of spherical particles on the surface of the granules as the transformation progresses. These spheres appear larger on more highly transformed materials.

**Table 2:** Conditions for granular synthesis of zeolite composites and subsequent weight fractions, *W*_f_, determined by Rietveld refinement of PXRD data, refinement conducted assuming sole presence of MOR, GIS and quartz (ESI 10). Duration of all treatments was 24 h

Material	Treatment	MOR *W*_f_	GIS *W*_f_
Mordenite	1.0 M NaCl^a^	0.92(1)	0.00(1)
Composite 1	0.7 M NaOH, 100 °C	0.91(2)	0.01(1)
Composite 2	0.8 M NaOH, 100 °C	0.85(1)	0.09(1)
Composite 3	0.9 M NaOH, 100 °C	0.62(1)	0.28(1)
Composite 4	1.0 M NaOH, 100 °C	0.57(1)	0.34(1)
Composite 5	1.1 M NaOH, 100 °C	0.25(1)	0.67(1)
Composite 6	1.2 M NaOH, 100 °C	0.22(6)	0.73(8)

a Activation by Na exchange.

### X-ray computed tomography (xCT) and local, image-guided diffraction

In order to probe the internal structure of the zeolite composite and the mechanism of transformation leading to the retention of particle morphology, xCT and local, image-guided diffraction were performed on three samples at the Dual Imaging and Diffraction (DIAD, K11) beamline at Diamond Light Source:^48^ activated (untransformed) mordenite, a MOR/GIS composite (Composite 4, 57 : 34 MOR : GIS ratio) and a further converted mordenite sample consisting of mostly zeolite P (Composite 5, 25 : 67 MOR : GIS ratio). A xCT field-of-view of 1.2 × 1.4 mm was used to image the granular composite and to guide the microdiffraction beam (25 × 25 μm) to regions of interest, such as the newly-formed crystallites of the surface, which can be isolated by ‘skimming’ the exterior shell of the particle. Alternatively, a beam trajectory through the centre of the particle would give a superposition of phases in both the outer shell and the interior. Tomography cross-sectional imaging of the untransformed, activated mordenite is displayed (Fig. 3h and l), alongside diffraction data collected at through point P (Fig. 3p). The tomography image shows little contrast within the interior of the particle, indicating uniform density and limited porosity throughout. Expectedly, the dominant reflections could be indexed to the MOR structure (Fig. 3p), though additional secondary phases assigned to quartz, analcime and magnetite were also observed (ESI 11†), consistent with laboratory powder diffraction data and as expected for a natural zeolite.

A cross-sectional tomography image for sample Composite 4 (Fig. 3i) suggests the presence of two main phases in the granule, with a lighter-contrast phase forming a ‘shell’ around the particle's interior. In line with SEM images (Fig. 3c), particles of spherical morphology are observed on the surface of the granule. Data collected by targeting the diffraction beam at the edge of the granule (point N in Fig. 3i) to collect a diffraction pattern for this ‘shell’ in isolation confirmed that GIS was the majority phase at the surface (Fig. 3n). A distinct ‘void’ region is present between the two phases, which is visible as darker contrast (Fig. 3j). The second phase is more uniform than the morphology observed in Composite 5, indicating that the transformation is incomplete. However, compared to the untransformed mordenite particle, there is increased texture to the interior including areas of porosity (darker areas in Fig. 3i), indicating that dissolution of the MOR phase is underway. Diffraction data collected from the centre of the particle (Fig. 3m) contained both the MOR and GIS phases with weight fractions of 0.75 and 0.18 respectively (ESI 11†); this is consistent with the idea of a ‘GIS shell’ encapsulating a mordenite interior. To further probe the spatial variation in phases to confirm this hypothesis, diffraction tomography data were collected on Composite 4. The intensity of key reflections for each phase (MOR (620) and GIS (200)) were integrated for each voxel, allowing for the reconstruction of the phase fractions of MOR or GIS as a 2D map. Fig. 3q shows a reconstructed slice through the centre of the particle. This confirms a higher intensity of the MOR phase at the centre of the granule with lower concentrations of GIS. Meanwhile, the shell of the granule contained large quantities of GIS at the expense of MOR, confirming the formation of a zeolite P shell during the transformation.

Significant morphological differences are observed in Composite 5 (Fig. 3g, k and o). The large, spherical sub-particles observed by SEM can be seen on the exterior of the granule in the tomography imaging, up to approximately 30 μm in diameter. Interestingly, the interior also contains similar sub-particles, albeit of only around 10 μm in diameter. Diffraction data collected at the point labelled R (Fig. 3r) confirms GIS as the dominant phase, with mordenite and quartz impurities (ESI 11†). This is in agreement with the bulk diffraction pattern (ESI 9†). We note that the diffraction beam is passing through the full particle, so the diffraction pattern (Fig. 3r) will contain phase information from all regions; thus, it is not possible from this data to determine whether the MOR phase remains concentrated in the centre from incomplete conversion of the particle or spread throughout. Based on these findings, we propose the macroscale mechanism of these transformations to be one of surface dissolution-recrystallisation, resulting in a dense ‘outer shell’ encapsulating a partially dissolved interior. Nucleation onto the parent material is likely preferred due to the elevated concentration of SDAs at the aluminous surface. The base then penetrates the outer shell, dissolving the interior, which then recrystallises into zeolite P (Fig. 3a).

### *In situ* image-guided diffraction studies of ion-exchange

**Fig. 4:**
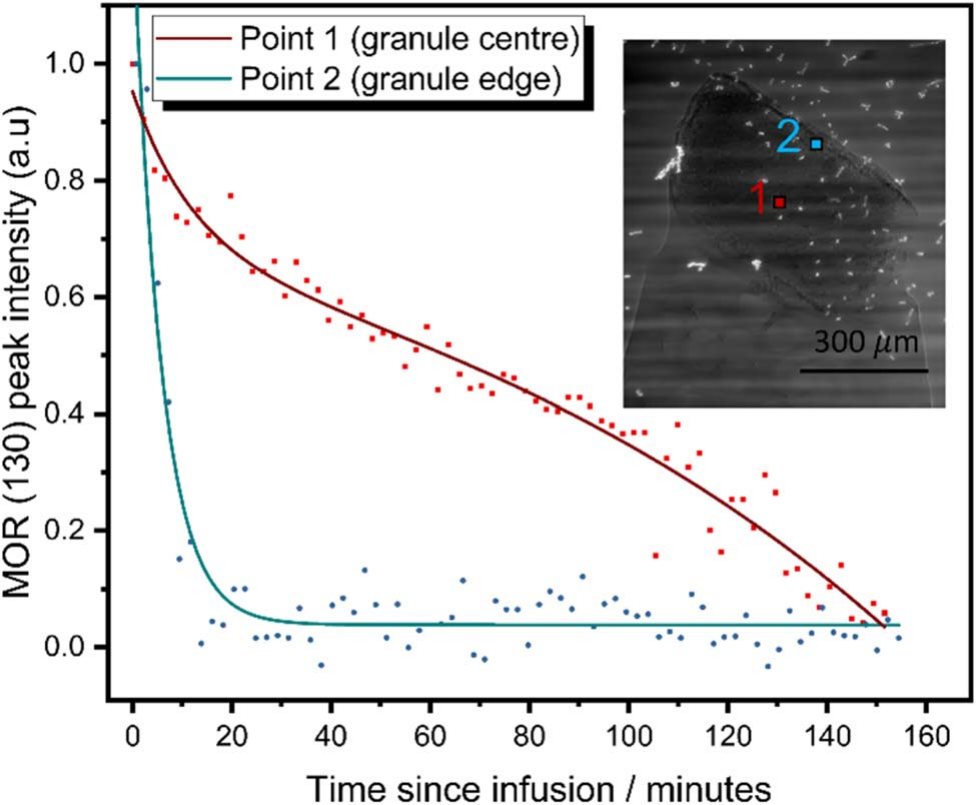
Spatially resolved MOR (130) reflection intensity as a function of time during *in situ* ion-exchange experiment. Composite 4 granule in 5000 ppm caesium solution. Inset: radiograph of mounted granule with annotated diffraction beam trajectories.

Given the high caesium affinity of the mordenite material compared to zeolite P observed in the batch ion-exchange experiments above, it is proposed that the mordenite core remains available and proficient in adsorbing caesium. However, xCT and local diffraction data (Fig. 3) suggest the core is encapsulated by the zeolite P shell, potentially restricting its accessibility. In order to establish the ability of the mordenite core to participate in ion-exchange processes, *in situ* image-guided diffraction caesium exchange experiments were performed. Two diffraction beam trajectories were selected to probe the outer mordenite core and the entire mordenite core (Fig. 4 inset). Exchange of caesium into the mordenite framework induces a change of space group from *C*2/*m* to C1.^57^ This change in symmetry results in changes to the diffraction pattern; for example, the (130) reflection is no longer observed (ESI 12†). Thus, the change in intensity of the reflection as a function of time during ion-exchange can be used to track the exchange of caesium at different positions within the composite granule. The relative intensities of the mordenite (130) reflection was monitored as a function of time (Fig. 4) after the granule was exposed to a 5000 ppm solution of Cs. The mordenite (130) reflection (*Q* = ∼1.07 Å^−1^) decreases in intensity in diffraction data collected at both the edge of mordenite core and the centre due to caesium incorporation within the framework channels. The MOR (130) reflection at the granule edge reaches <5% of the original intensity within 30 minutes of ion-exchange, whereas the granule centre sees significantly slower exchange, with a reduction to <5% of the original intensity not reached until 150 minutes. Critically, these results suggest that the zeolite P shell is largely permeable and that the mordenite core retains the ability to function as an ion-exchanger after partial transformation, with the outermost mordenite accessible by the solution almost instantaneously. The integrated intensity of the GIS (200) reflection from the outer shell, which similarly decreases in intensity upon caesium uptake, was also monitored as a function of time (ESI 12†). The reduction in intensity is slower than the outer mordenite core, despite being on the surface of the granule. This is potentially indictive of a faster rate of caesium uptake in the mordenite core compared to the zeolite P shell. Given the high selectivity for caesium exhibited by mordenites, we therefore expect the mordenite core to play a key role in caesium abatement from industrially-relevant, low concentration solutions. Thus, the dual-phase nature of the composites is key to their functionality. X-ray CT of the granule before and after ion-exchange show no significant changes to the granule microstructure after ion-exchange (ESI 12†).

### Rapid ion-exchange (RIX) active flow trials

**Fig. 5:**
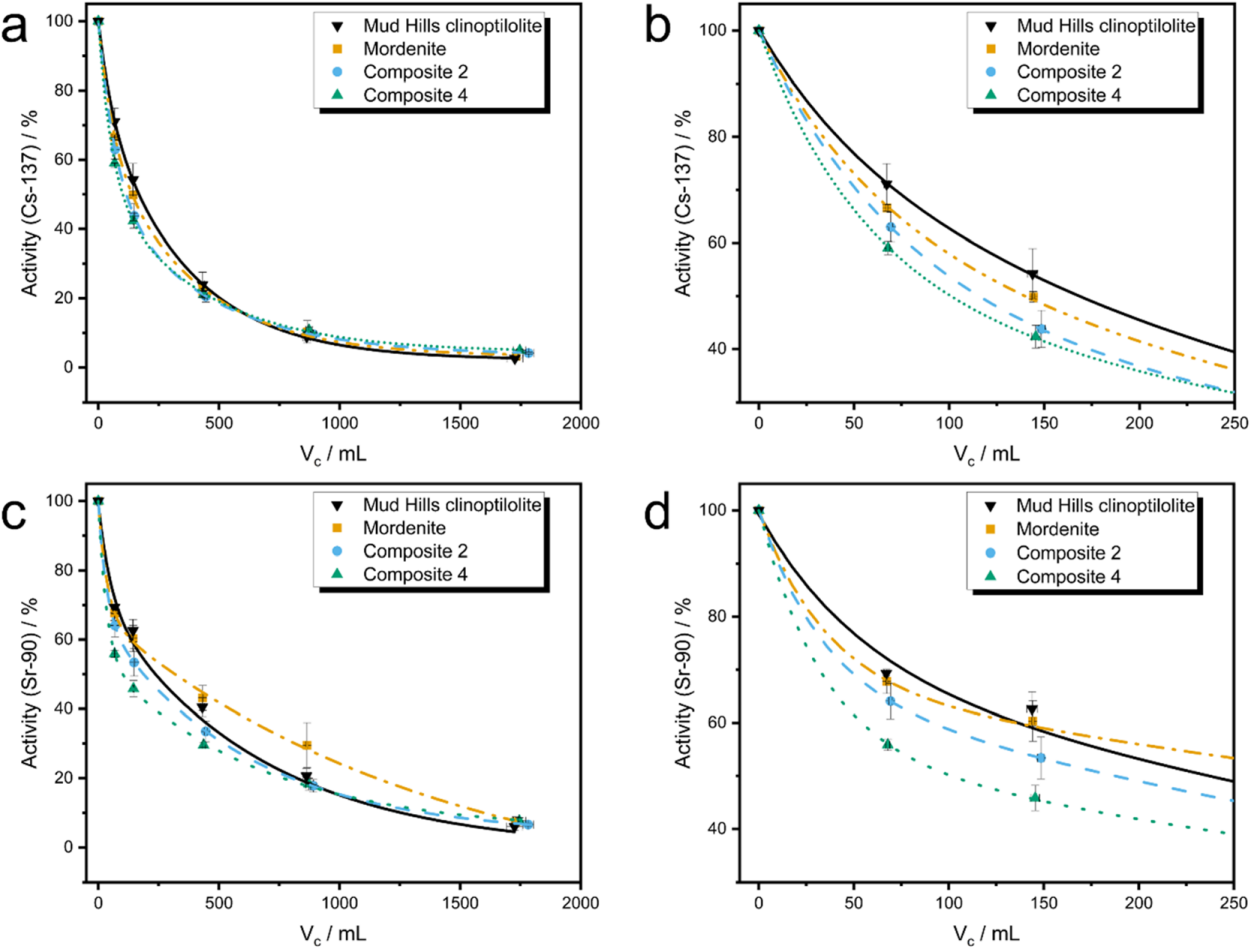
RIX uptake data for Mud Hills clinoptilolite, mordenite, Composite 2 and Composite 4. (a) Cs-137 activity throughout duration of experiment. (b) Cs-137 activity during initial stages of experiment (first 250 mL). (c) Sr-90 activity throughout duration of experiment. (d) Sr-90 activity during initial stages of experiment (first 250 mL).

Industrial effluent residence times within columns can be as short as a few minutes, making ion-exchange kinetics crucial to successful remediation.^17^ In order to probe the kinetics of ion-exchange within the developed granular composites, in addition to their physical suitability in column systems and, therefore, assess possible use in an industrial setting, newly developed dynamic flow tests (rapid ion-exchange (RIX) method), were conducted. Solutions containing Cs-137 and Sr-90 radionuclides were used in conjunction with Composites 2 and 4 (85 : 9 and 57 : 34 MOR : GIS ratio by weight, respectively), in addition to an untransformed sample of mordenite and Mud Hills clinoptilolite, both of which were also sieved to 400–600 μm particle size (see Methods). The use of radionuclides facilitated accurate analysis of the low concentrations typically observed in the SIXEP feed stream (Cs ∼0.017 ppm, Sr ∼0.0005 ppm)^6^ which are too low to be probed using non-active methods such as ICP-MS. Cs-137 and Sr-90 uptake curves for the materials are displayed in Fig. 5. The fit of the two-parameter exponential decay functions to the Cs-137 data are generally excellent with all *R*^2^ values above 0.993 (exponential parameters and fitting data for each material is available in the ESI 13†). Fits to Sr-90 data are also generally good, with *R*^2^ values typically greater than 0.980. The slightly poorer fits to Sr-90 data caused by the greater variation in results is likely due to the difference in counting methods (gamma *versus* Cherenkov) and the lower activity levels of Sr-90 in the initial solution.

The ability of the material to remove ions from the reservoir was calculated from the rate that the activity (number of decays per second) in the reservoir decreased after a given volume of active simulant (*V*_c_) had passed through the material; the lower the activity of a given volume, the more radionuclides the material has sorbed. Because the active simulant liquor was pumped through the column and recirculated back into the liquor reservoir, the radionuclides become more dilute within the solution as the experiment progresses, which will reduce the rate of removal. This can be corrected for to give the probability of sorption at a given volume (*α*), which can be estimated using eqn (3) (*V*_r_ = reservoir volume, *V*_f_ = flow rate, *C* = concentration of reservoir for a particular species, see ESI 13† for derivation). A summary of sorption probabilities at three volume intervals (50, 300 and 1500 mL) is provided in Table 3.3
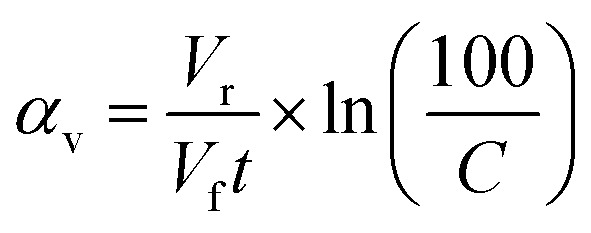


**Table 3:** Probability of sorption (*α*_V_) at various volumes (V cm^−3^) for Cs-137 and Sr-90 for Mud Hills clinoptilolite, mordenite, Composite 2 and Composite 4

Material	*α*_50(Cs)_	*α*_300(Cs)_	*α*_1500(Cs)_	*α*_50(Sr)_	*α*_300(Sr)_	*α*_1500(Sr)_
Mud Hills clinoptilolite	0.53	0.36	0.23	0.53	0.26	0.18
Mordenite	0.63	0.38	0.21	0.60	0.23	0.14
Composite 2 (85 : 9 MOR : GIS ratio)	0.70	0.42	0.20	0.74	0.29	0.16
Composite 4 (57 : 34 MOR : GIS ratio)	0.83	0.42	0.19	0.98	0.34	0.16

Both Mud Hills clinoptilolite and the activated, untransformed mordenite display very similar Cs-137 uptake curves, in agreement with the good Cs sorption behaviour observed in earlier powder capacity studies, and with previous literature.^18^ The slightly higher initial uptake of Cs in mordenite compared to Mud Hills might reflect the enhanced ionic diffusion through larger 12 membered rings compared to 10 membered rings in the HEU structure, although small changes in particle permeability may also affect this (see Mud Hills clinoptilolite xCT, ESI 14†). Both composites show faster initial uptake of Cs compared to both Mud Hills and the parent mordenite; this is reflected in the higher initial *α*_50_ values (0.70 and 0.83 *vs.* 0.53 and 0.63, for Composite 2, Composite 4, Mud Hills and mordenite, respectively). This is unlikely to originate from enhanced ionic diffusion within the newly-formed GIS zeolite crystal structure, given that the GIS framework contains relatively narrow 8- and 4-membered ring units. Instead, this is likely to reflect the enhanced diffusion through the mordenite phase thanks to desilication and microscopic diffusion through the more porous composite (clearly observed in Fig. 3h–k), both features are induced by the transformation.

The initial uptake rate of Sr-90 in the mordenite material is higher than Mud Hills clinoptilolite (*α*_50_ of 0.60 and 0.53, respectively). However, after ∼150 mL throughput, the rate of uptake for mordenite significantly reduces and remains lower than for Mud Hills clinoptilolite throughout the experiment. Composites 2 and 4 showed significantly enhanced uptake of Sr-90 at low volumes compared to both naturally sourced analogues resulting in the trend: Composite 4 > Composite 2 > Mud Hills clinoptilolite > mordenite. The is likely to originate from both the enhanced strontium capacity, as revealed through adsorption isotherms, and the increased surface area and porosity, which can be observed in xCT images (Fig. 3h–k); this allows easier access of ions to surface adsorption sites, an effect which is likely to be significant for the larger hydrated strontium ion when compared to caesium.

The composites continue to show increased uptake of strontium compared to the parent mordenite for all throughput volumes used in these experiments. These trends are also supported by batch kinetic studies conducted on powdered composites (ESI 15†). Around 1000 mL of throughput, Mud Hills clinoptilolite surpasses the uptake capacity of the composites. This perhaps marks the point at which the majority of surface adsorption sites are taken up, and diffusion through the bulk of the material becomes the limiting factor. At this point, although the GIS phase has significantly higher capacity than either HEU or MOR, the small 8 MRs will slow ion diffusion. It is noteworthy that all four Sr curves remain further from equilibrium compared to the caesium analogues at the end of the experiment, likely due to the slower movement of the large, hydrated, divalent strontium ion. This is consistent with both complimentary batch kinetic studies (ESI 15†) and literature.^18^

## Discussion

The cost, availability and deployment-ready morphology of natural zeolites are attractive features for a range of industrial processes. However, the limited number of frameworks found naturally prohibits more widespread use and leads to overreliance on specific natural sources, with little flexibility to tailor functionality to specific chemical conditions. Whilst minor modification to Si/Al ratio and textural properties using low temperature base treatments can result in minor changes to physical properties, our results demonstrate that higher temperatures drive controllable structural transformations which diversify the chemistry observed from a natural zeolite. Despite mordenite's excellent natural affinity for caesium^18^ and high geological abundance^58^ its poor strontium uptake would typically discount it from consideration as a dual-ion Cs/Sr adsorber for deployment within the nuclear industry. The partial transformation of a naturally sourced mordenite into zeolite P leads to a significant improvement in Sr-uptake in batch ion-exchanges in the presence of competitive cations which, critically, was not at the expense of caesium uptake, which remained high. As a result, the composite containing a 40 : 60 mordenite : zeolite P phase ratio shows comparable dual-uptake in batch ion-exchange experiments, over double the strontium capacity and comparable caesium capacity to the current industry standard, Mud Hills clinoptilolite. Significantly, the origin of the improved dual ion-exchange behaviour is not simply the presence of two materials with complementary ion-exchange chemistry; a 50 : 50 mixture of mordenite and zeolite P showed poorer strontium uptake in batch ion experiments than composites with similar phase ratios. Instead, we propose that the combination of having both the more aluminous zeolite P framework and the concurrent partial desilication of the mordenite framework result in the increase in strontium affinity with little loss of caesium affinity. This demonstrates that unique ion-exchange chemistry is afforded by the partial inter-zeolite transformation.

The choice of the base concentration affords an extraordinary degree of control over the phase ratio, in this case the ratio of mordenite and zeolite P phases; this is achieved over the full interconversion phase range. The impact of this is two-fold: firstly, this process serves to diversify the chemistry of natural zeolites, meaning that both the library of potential natural sources for ion-exchangers, and the potential applications for natural zeolites are vastly widened. Secondly, the fine chemical control over the phase composition delivers materials with excellent discrimination between uptake of strontium and caesium allowing the overall ion-exchange properties to be finely, and predictably, tuned. This opens up the possibility that ion-exchange properties of mineral-source-derived composites could be designed for a given waste-stream.

The ability to readily generate column-ready, morphologically-controlled granular composites in a simple, low-cost, one pot method, whilst retaining the advantages of natural zeolites over synthetic sources, adds industrial appeal to this process. The inter-zeolite transformations core–shell morphologies from a low-grade source, without the need for additional components (*e.g.* binders) or processing that would be required for other mixed phase systems, such as a 50 : 50 mix of parent mordenite and fully converted zeolite P, which also exhibits poorer affinity for strontium. The crucial retention of the size of the parent particles in the dual-phase composites is likely the result of the dense outer shell of zeolite P which initially forms on the surface of granules during the transformation. Our combined SEM/xCT/XRD characterisations show that this shell is present in the partially transformed materials and remains even when the transformation is close to complete, with more porous areas forming in the centre of the composites. *In situ* xCT/XRD experiments during ion-exchange demonstrate that the core remains accessible to the caesium. In addition to the deployment advantages of maintained granularity, our RIX measurements highlight that the partially transformed granular composites demonstrate a remarkable improvement in both Cs-137 and Sr-90 uptake rates compared to the initial mordenite system. In fact, this enhancement is to such an extent that the granular composites exhibited superior Cs-137 and Sr-90 uptake rates when compared to industry standard, Mud Hills clinoptilolite, either because of the altered ion-exchange properties of the phases after the partial transformation, or because of the difference in porosity results in improved ion diffusion into the particles. These results open up inter-zeolite transformation as a route to more efficient waste processing, in addition to the potential to design the adsorber's morphology for a particular column system and contact time. We note that when selecting a material to deploy in nuclear applications, it is important to consider both the mechanical properties of the material and its impact on the cost of material immobilisation, medium-long term storage in a radioactive waste store and eventual disposal in a geological repository; these factors will be explored in future work. Taken together, the chemical and morphological tunability of the process presented here offer a straightforward and cost-effective way to tune both the thermodynamics and kinetics of ion-exchange, opening up the possibility of “designer” composites tailored towards specific effluent streams and systems. Beyond nuclear waste management, species selectivity is crucial to many applications of zeolites across the chemical sciences. Granular zeolite composites with tuneable chemical functionality and morphology may find ready application in areas where there are multiple species of interest, for example water purification, multi-gas absorbers,^59,60^ multi-molecular separators^61^ or multi-process catalysts,^62^ or where the combination of the complementary characteristics of different frameworks (*e.g.* species selectivity and adsorption capacity) may prove advantageous. While our focus here has been on extending the functionality of a natural zeolite, similar control over partial transformations should be possible in synthetic zeolite monoliths allowing the rich vein of inter-zeolite transformation chemistry^40^ to be used for the production of tuneable composite particles.

## Conclusions

This study has revealed new insights into the interzeolite transformation of natural zeolites. Chemically-controlled partial inter-zeolite transformation has been demonstrated as a method to obtain dual-phase zeolite composites where the atomic phase and particle morphology are simultaneously controlled. Batch- and rapid-ion-exchange experiments and adsorption isotherms reveal aspects of the dual-ion (Cs/Sr) uptake characteristics of dual phase mordenite/Na–P composites synthesised from a low-grade natural mordenite source: composites exhibit significantly increased ion-exchange affinity for strontium compared to the parent mordenite whilst retaining high affinity for caesium. Notably, the strontium uptake of composites was higher than for a physical mixture of the mordenite/zeolite P end-members materials with similar phase ratios; this was assigned to the role of partial desilication of the mordenite framework in addition to the formation of the more aluminous zeolite P phase. A 40 : 60 phase-ratio composite achieving Cs/Sr uptakes comparable, and uptake rates surpassing Mud Hills clinoptilolite. Scanning electron microscopy (SEM), X-ray computed tomography (xCT) and image-guided micro-X-ray diffraction reveal the core–shell structure of the composites and further allows us to propose a formation mechanism for granular composites and, hence, the origin of the morphological control. *In situ* ion-exchange experiments show that Cs uptake is rapid at the edge of the mordenite core, demonstrating that the dual-phase nature of the composites leads to advantageous ion-exchange capabilities. These results show that partial interzeolite transformation can diversify and tune the chemistry of natural zeolites whilst, importantly, also retaining their particle size and morphological advantages for industrial deployment.

## Data availability

Data related to this article is available at https://doi.org/10.25500/edata.bham.00001139.

## Author contributions

J. R., P. A. and J. H. designed the powder synthesis, batch ion-exchange testing and formation of granular composites experiments. A. J., J. R., P. A. and J. H. designed and ran the X-ray computed tomography (xCT) and local, image-guided diffraction experiments; J. R. and A. J. processed and interpreted the data. T. C., J. R., N. F., P. A. and J. H. designed the RIX active flow trials experiments. J. R. ran the powder synthesis, batch ion-exchange testing and formation of granular composites experiments and processed and interpreted the data (with guidance from P. A. and J. H.). J. R., P. A., J. H. and A. N. designed and performed the *in situ*, image-guided diffraction ion-exchange experiments. J. R. and N. F. performed the RIX active flow trials experiments and processed and interpreted the data (with guidance from T. C.). P. A. undertook analysis to estimate the extent of desilication of the mordenite in the transformation process (ESI 4†). J. R. designed, executed the experiments, and processed the data, for the adsorption isotherm studies (ESI 7†) J. R. designed, executed the experiments, and processed the data, for the batch rate studies (ESI 15†). A. F. obtained selected SEM images. H. F. performed gas adsorption experiments and processed the associated data. S. K. provided guidance throughout the work. J. R. produced all figures and compiled the manuscript with significant contributions from P. A., J. H., A. J. and T. C.

## Conflicts of interest

There are no conflicts to declare.

## Supplementary Material

SC-015-D4SC02664K-s001
