# Supplementary material for: Engineered species-selective ion-exchange in tuneable dual-phase zeolite composites
Source: Chem Sci. 2024 Aug 1;15(34):13699–711. doi: 10.1039/d4sc02664k (PMC11352454; doi:10.1039/d4sc02664k)
Supplement: SC-015-D4SC02664K-s001 [file SC-015-D4SC02664K-s001.pdf]

Electronic Supplementary Material (ESI) for Chemical Science.
This journal is © The Royal Society of Chemistry 2024

Supplementary Information for

Engineered species-selective ion-exchange in tuneable dual-phase zeolite composites

James L. A. Reed[a], Andrew James[b], Thomas Carey[c], Neelam Fitzgerald[c], Simon Kellet[d], Antony Nearchou[a], Adele L. Farrelly[a], Harrison A. H. Fell[a], Phoebe K. Allan[a]* and Joseph A. Hriljac[b]*

[a]School of Chemistry, University of Birmingham, Birmingham, UK. [b]Diamond Light Source Ltd., Harwell Science and Innovation Campus, Didcot, UK. [c]National Nuclear Laboratory, Springfields site Ltd, Salwick, Preston, UK. [d]Sellafield Ltd, Sellafield, Seascale, Cumbria, UK.

Contents

1. Extra information regarding DIAD beamline calibration, mounting, positioning, alignment and data analysis pipelines 3

2. Powder XRD patterns of zeolite composites 4

3. Rietveld refinements of powder zeolite composites 5

4. Desilication estimation 12

5. Porosimetry measurements 13

6. Peak width fitting 14

7. Adsorption studies 15

8. Non industrially deployed ion-exchange materials 18

9. Granular PXRD patterns 19

10. Rietveld refinements of granular zeolite composites 20

11. Rietveld refinements of local diffraction patterns of granules 25

12. In-situ image-guided diffraction studies of ion-exchange 28

13. Rapid ion-exchange uptake curve fitting and derivation 30

14. xCT of Mud Hills clinoptilolite 32

15. Batch rate studies 33

16. References 36

1. Extra information regarding DIAD beamline calibration, mounting, positioning, alignment and data analysis pipelines

Specimen mounting, positioning and alignment

Specimens were mounted on the top of a short length of 0.5 mm diameter polyamide tubing using Araldite two-part setting resin. The tubing was then attached to a 3 mm Huber goniometer mounting pin and placed on a Huber 1005 manual goniometer stage. This stage was then placed onto the General Tomography Stage (GTS) on the instrument. The GTS stage facilitated specimen alignment to the rotation axis (using translational stages) and provided rotation during the tomographic measurements.

Data Analysis (automated and manual)

Automatic tomographic reconstruction for the imaging data was provided through a configured SAVU plugin chain consisting of the steps outlined in Table T1. Automated reduction of 2D diffraction data to 1-dimension (I vs. Q) plots was configured through DAWN.

Manual tomographic reconstruction of the diffraction data proceeded after integration of the diffracted intensity between the following q-ranges from the I vs. q plots: 2.151 Å-1 – 2.188 Å-1 for Mordenite; 1.220 Å-1 – 1.276 Å-1 for Na-P (GIS). Sinograms of the integrated intensity values were reconstructed using the simultaneous iterative reconstruction technique (SIRT) with the algorithm provided as part of the ASTRA toolbox[1], with 25 iterations for single slice reconstructions and 50 iterations for the multi-slice reconstruction. A consequence of the correlated imaging and diffraction system was that the rotation centre setting was provided by the Vo-centring[2] plugin reported in the imaging tomography. Note, individual diffraction frames were not corrected for changes in the sample to detector distance, as a function of the rotation angle, though possible using the tomography data, the size of the specimens was sufficiently small to warrant neglecting this for this preliminary study.

Table 1: Automated and Manual data analysis steps and settings used for data processing.

Automated Imaging Pipeline Implemented in SAVU	1.Apply Dark and flat field correction
	2.Determine centre of rotation using Vo centring using search range
	3. Reconstruct using Filtered Back Projection from the ASTRA toolbox
Automated Diffraction Data Reduction Pipeline	1. Import nearest diffraction geometry description
	2. Mask bad pixels
	3. Reduce to I vs q using the following settings: a. Qmin= 0.97 $Å^{-1}$, Qmax = 10 $Å^{-1}$ b. nbins=3500 (dq =0.00258 $Å^{-1}$) c. Pixel splitting
	4. Integrate regions corresponding to the peak positions of important phases
	5. Convert to I vs 2theta, and export for further analysis.
Manual Diffraction Tomography Pipeline	Pipeline 1. Reconstruct sinograms from D4 above using SIRT algorithm implemented in the ASTRA toolbox[1] using the following settings: a. Number of iterations: 25 (single slice), 50 (long scan) b. Centre of rotation determined from imaging (output of step 2).

2. Powder XRD patterns of zeolite composites

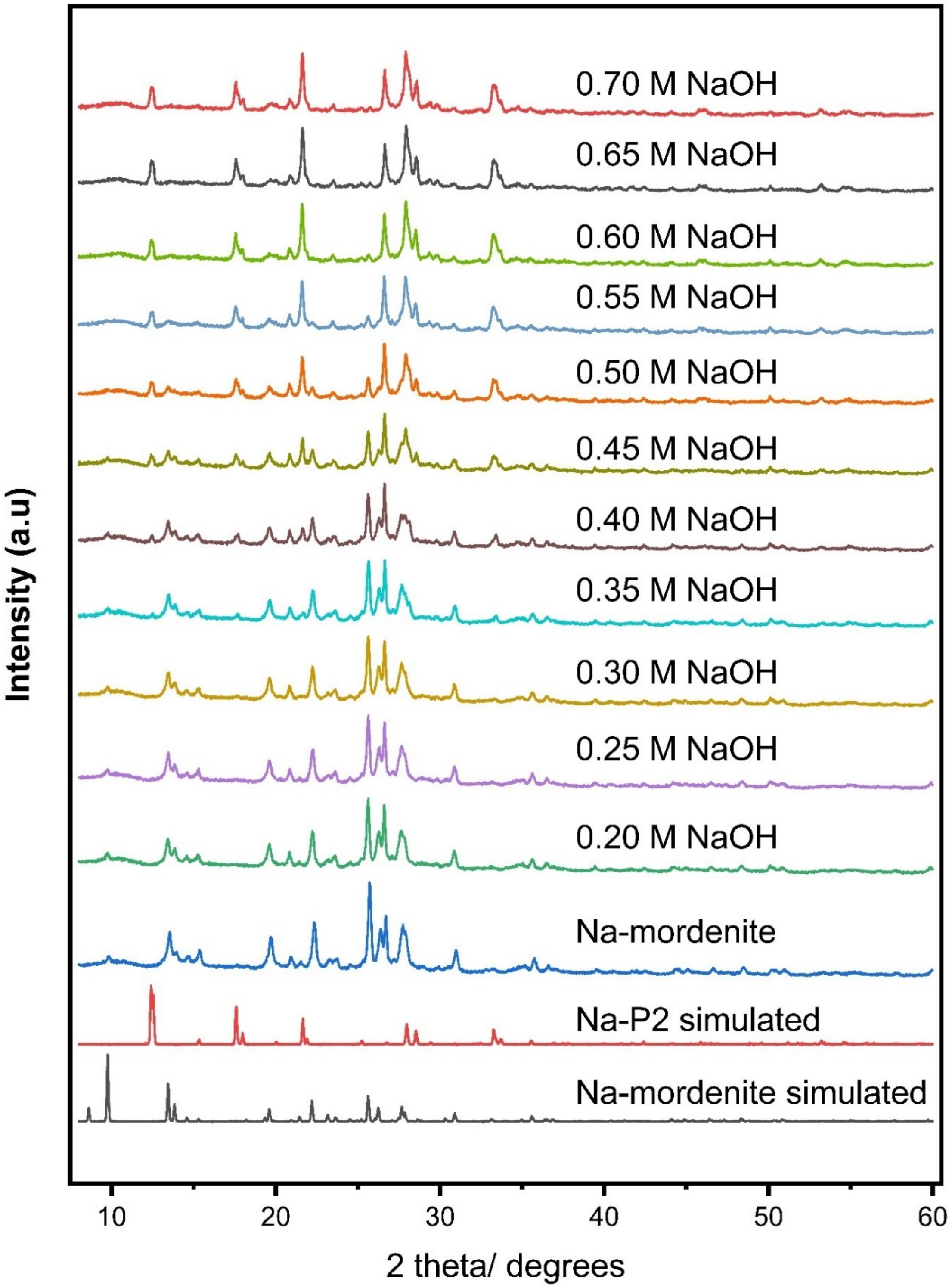

Fig.1 PXRD data for all zeolite composite materials derived from mordenite. NaOH concentrations are labelled in addition to simulated patterns for both Na-mordenite and Na-P2 being displayed (λ = 1.5406 Å).

3. Rietveld refinements of powder zeolite composites

Refinements were completed using GSAS ii[3] assuming the sole presence of three phases: Na-MOR, Na-P2 and quartz. A summary of fit qualities and weight fractions is shown in Table 2.

Na-mordenite

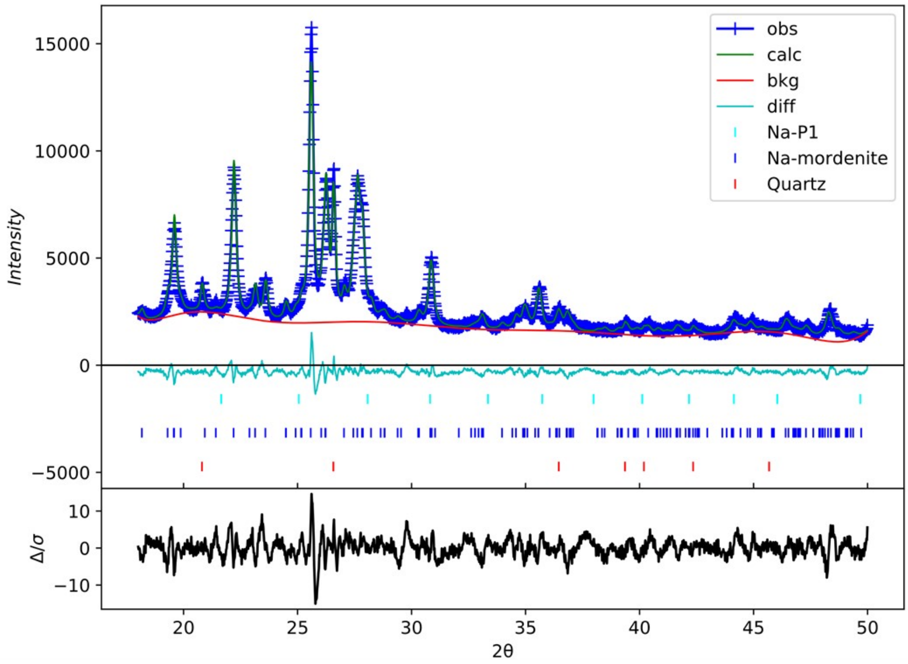

Fig. 2 Rietveld refinement of PXRD data for Na-mordenite.

0.2 M NaOH

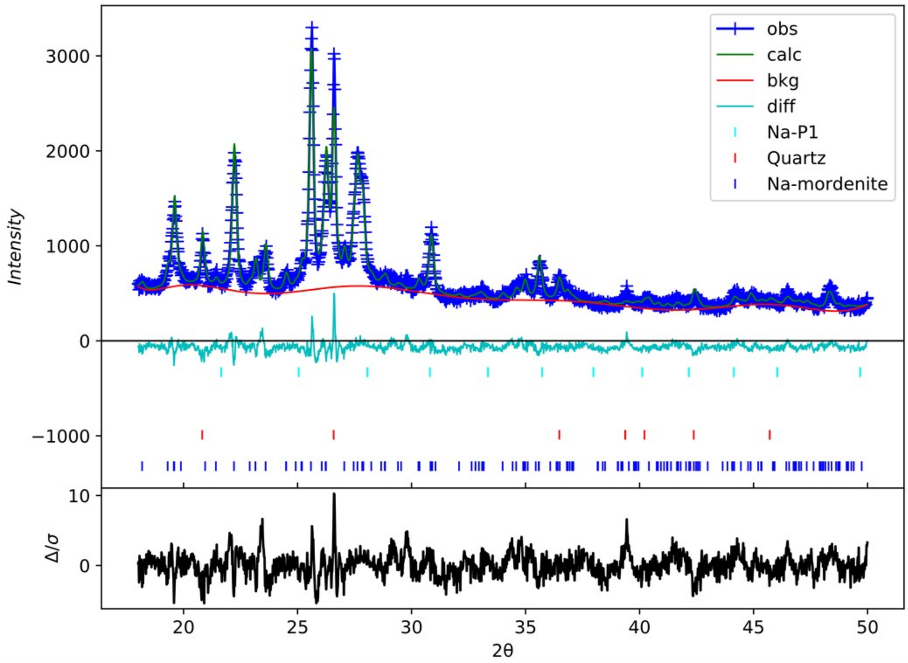

Fig. 3 Rietveld refinement of PXRD data for 0.20 M NaOH treated mordenite.

0.25 M NaOH

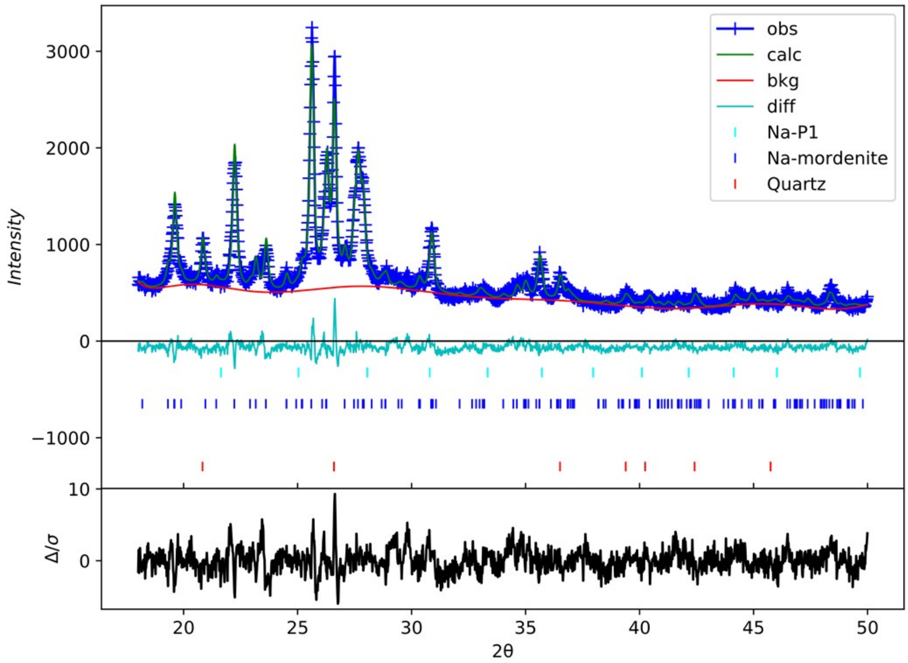

Fig. 4 Rietveld refinement of PXRD data for 0.25 M NaOH treated mordenite.

0.30 M NaOH

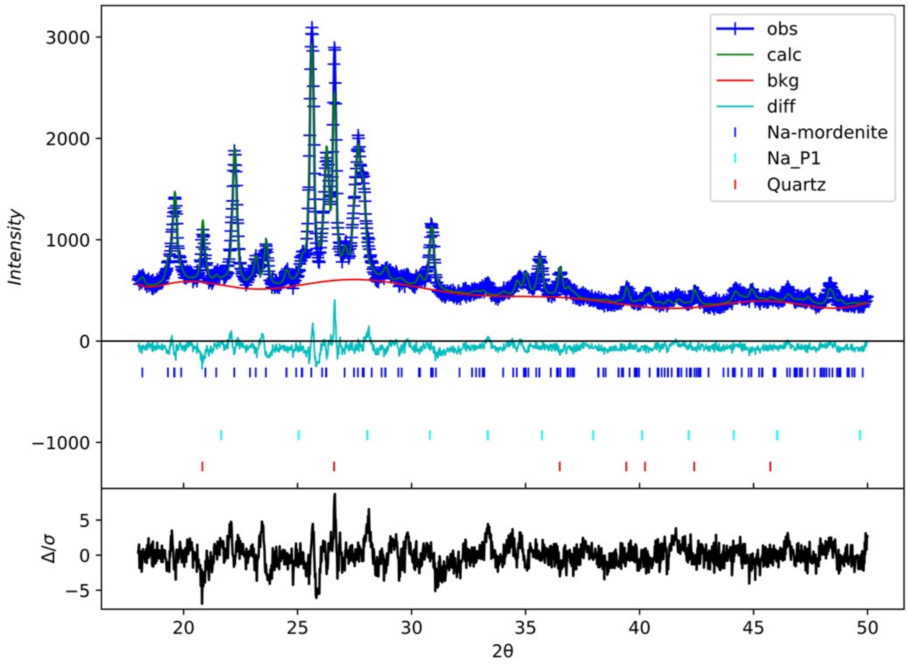

Fig. 5 Rietveld refinement of PXRD data for 0.30 M NaOH treated mordenite.

0.35 M NaOH

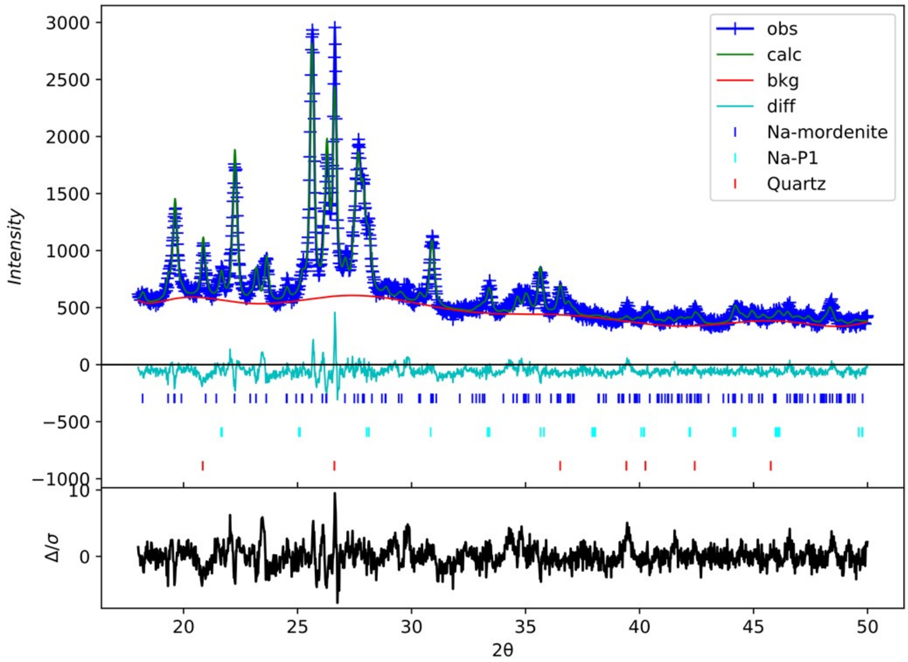

Fig. 6 Rietveld refinement of PXRD data for 0.35 M NaOH treated mordenite.

0.40 M NaOH

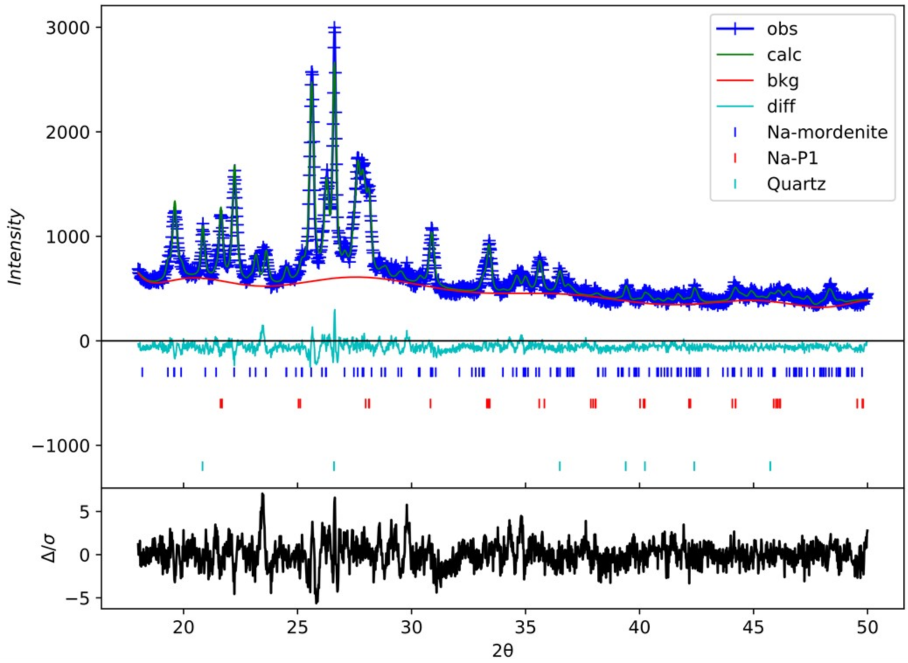

Fig. 7 Rietveld refinement of PXRD data for 0.40 M NaOH treated mordenite.

0.45 M NaOH

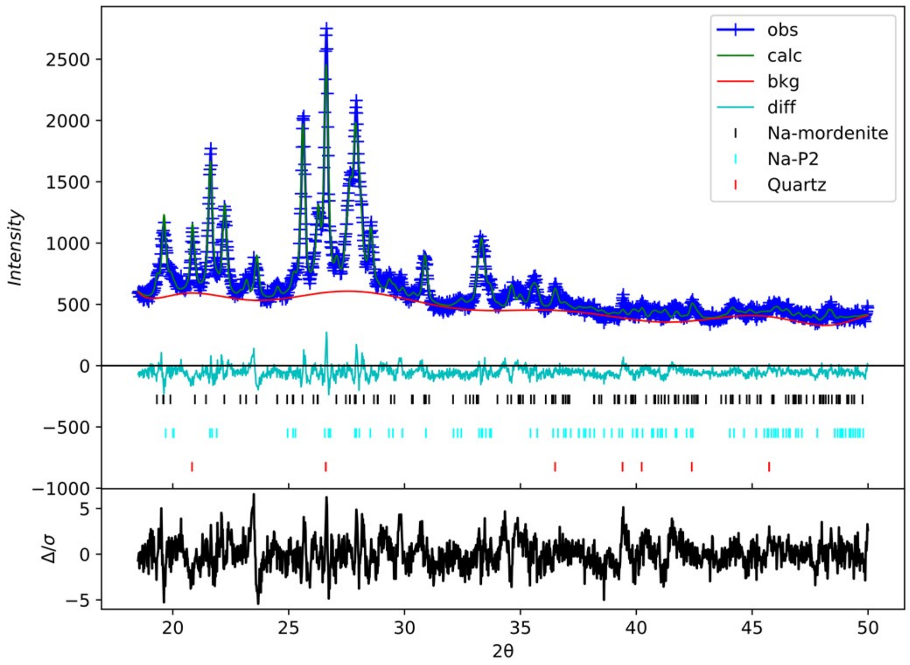

Fig. 8 Rietveld refinement of PXRD data for 0.45 M NaOH treated mordenite.

0.50 M NaOH

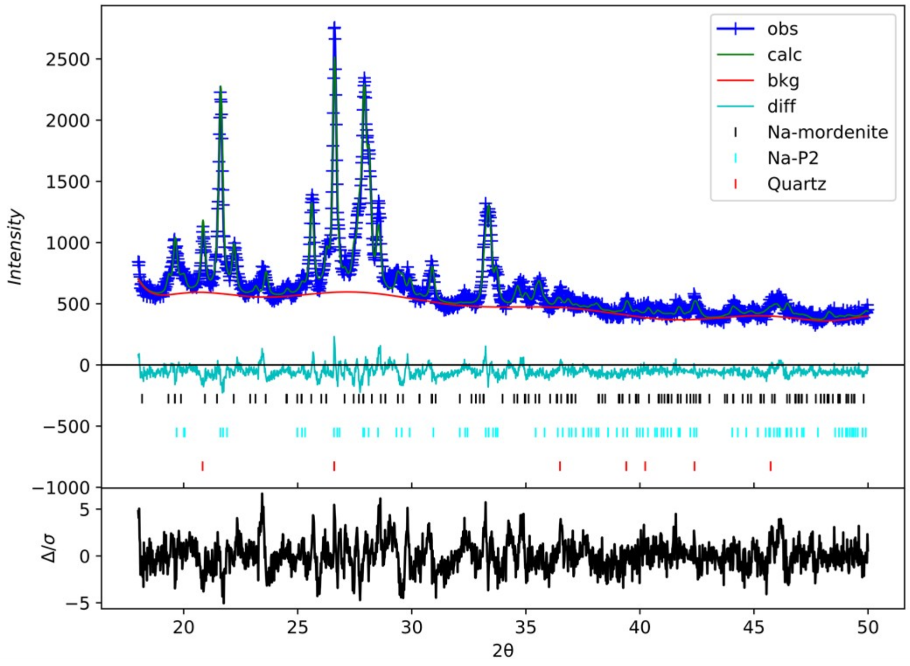

Fig. 9 Rietveld refinement of PXRD data for 0.50 M NaOH treated mordenite.

0.55 M NaOH

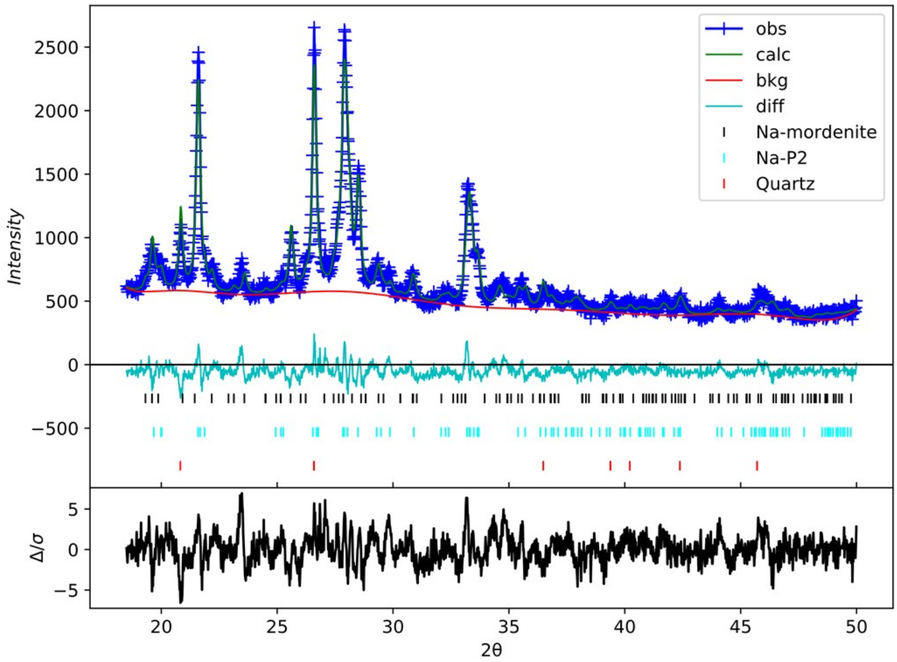

Fig. 10 Rietveld refinement of PXRD data for 0.25 M NaOH treated mordenite.

0.60 M NaOH

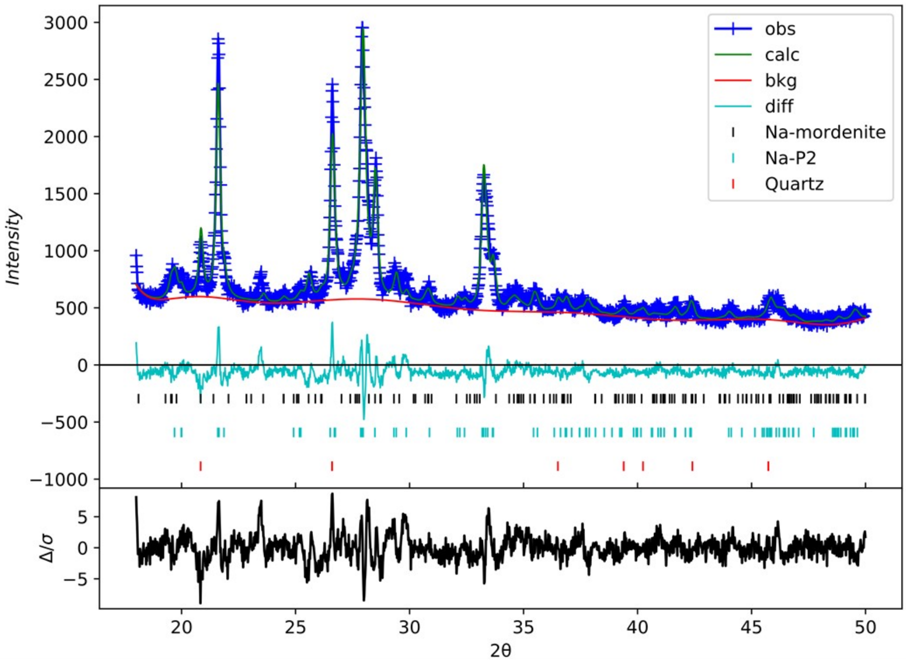

Fig. 11 Rietveld refinement of PXRD data for 0.60 M NaOH treated mordenite.

0.65 M NaOH

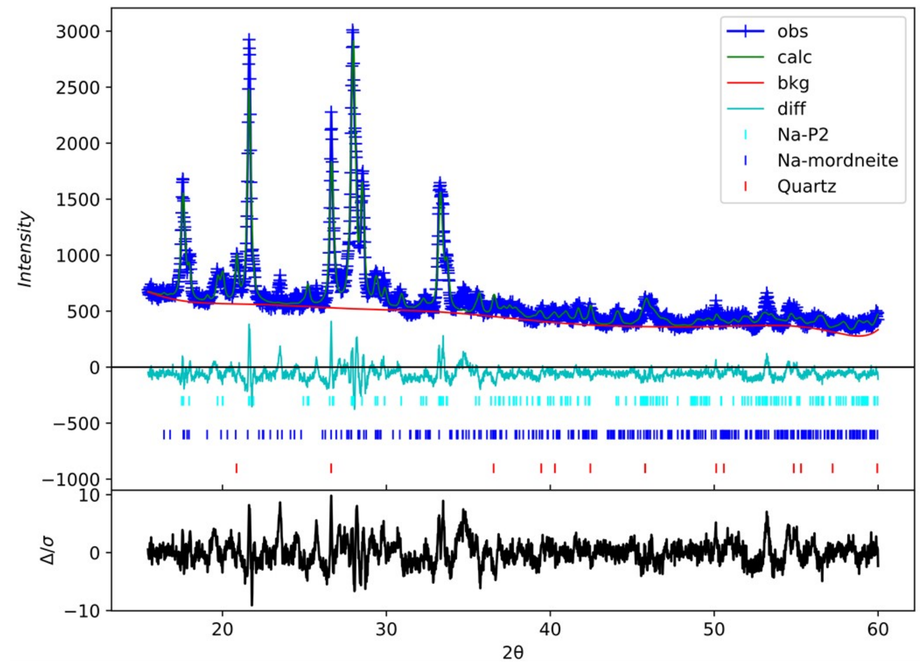

Fig. 12 Rietveld refinement of PXRD data for 0.65 M NaOH treated mordenite.

0.70 M NaOH

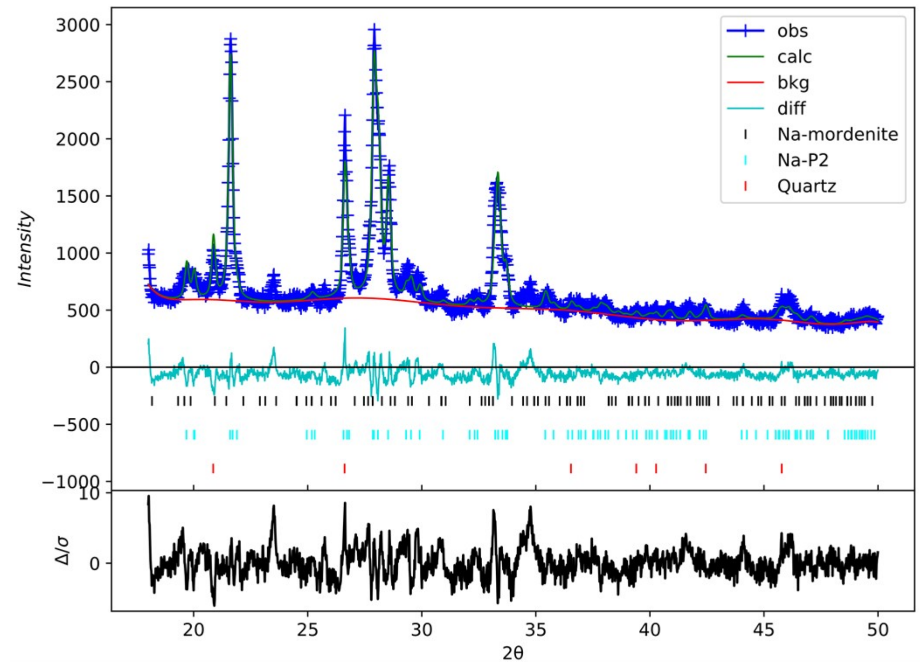

Fig. 13 Rietveld refinement of PXRD data for 0.70 M NaOH treated mordenite.

Table 2: Summary of refinement results for powdered materials.

Treatment	wR / %	GOF	MOR W_f	GIS W_f	Quartz W_f
None (Na-mordneite)	4.89	2.51	0.87(2)	0.00(1)	0.13(1)
0.20 M NaOH	6.63	1.65	0.92(1)	0.00(1)	0.08(1)
0.25 M NaOH	6.38	1.60	0.93(1)	0.00(1)	0.07(1)
0.30 M NaOH	6.42	1.61	0.95(1)	0.00(1)	0.05(1)
0.35 M NaOH	6.47	1.63	0.84(1)	0.06(1)	0.10(1)
0.40 M NaOH	5.89	1.48	0.76(1)	0.14(1)	0.11(1)
0.45 M NaOH	6.20	1.57	0.61(1)	0.29(1)	0.10(1)
0.50 M NaOH	6.39	1.61	0.32(1)	0.56(1)	0.12(1)
0.55 M NaOH	6.86	1.72	0.25(1)	0.65(1)	0.11(1)
0.60 M NaOH	7.57	1.92	0.15(1)	0.74(1)	0.11(1)
0.65 M NaOH	8.45	2.07	0.03(1)	0.89(1)	0.08(1)
0.70 M NaOH	7.89	2.00	0.00(1)	0.93(1)	0.07(1)

4. Desilication estimation

The change in the mordneite's Si:Al ratio can be estimated using the weight fractions (W_f's) from the Rietveld refinemnets by assuming that all newly-crystalised zeolite P has the same Si:Al ratio (2.5) as the end member, which depicts a 'complete' transformation. Theoretical Si:Al ratios have been calculated assuming the end-member Si:Al ratios (4.59 and 2.49 for mordenite and zeolite P) remain constant throughout (Fig. 14a). During the transformation period (0.30 -0.60 M), The theroertical Si:Al ratio is consistantly higher than the observed, suggesting that desilication of the parent mordenite is occuring in the transformation. The estimated mordenite Si:Al ratio can be calculated for each data point (Fig. 14b).

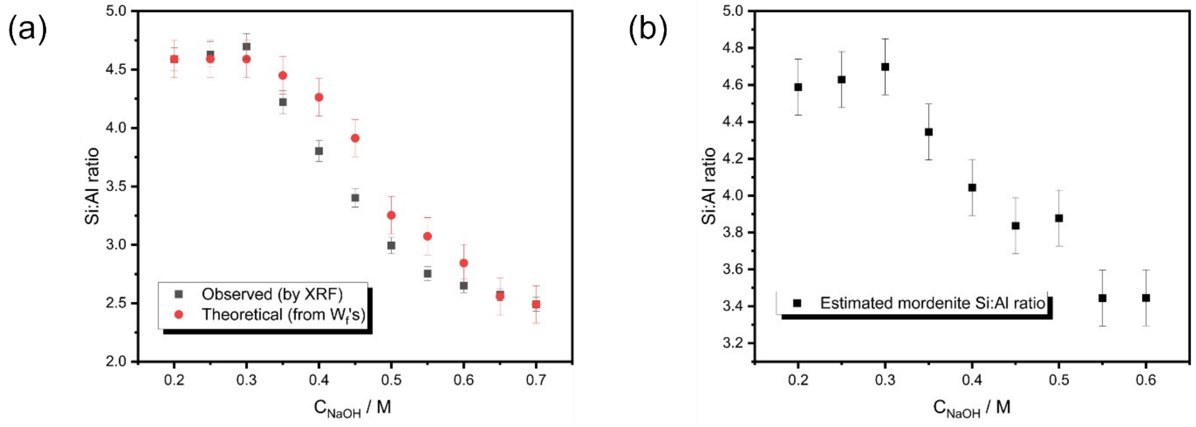

Fig. 14 Si:Al ratio during the transformation (a) Observed Si:Al ratio and theoretical Si:Al ratio plotted as a function of NaOH concentration. (b) Estimated mordenite Si:Al ratio as a function of NaOH concentration.

5. Porosimetry measurements

Porosimetry measurements were carried out using a Micrometrics 3Flex volumetric gas sorption analyser at 77 K. BET surface areas were calculated by applying the Rouquerol correction to select the range $p/p_0 = 0.01 – 0.05$[4].

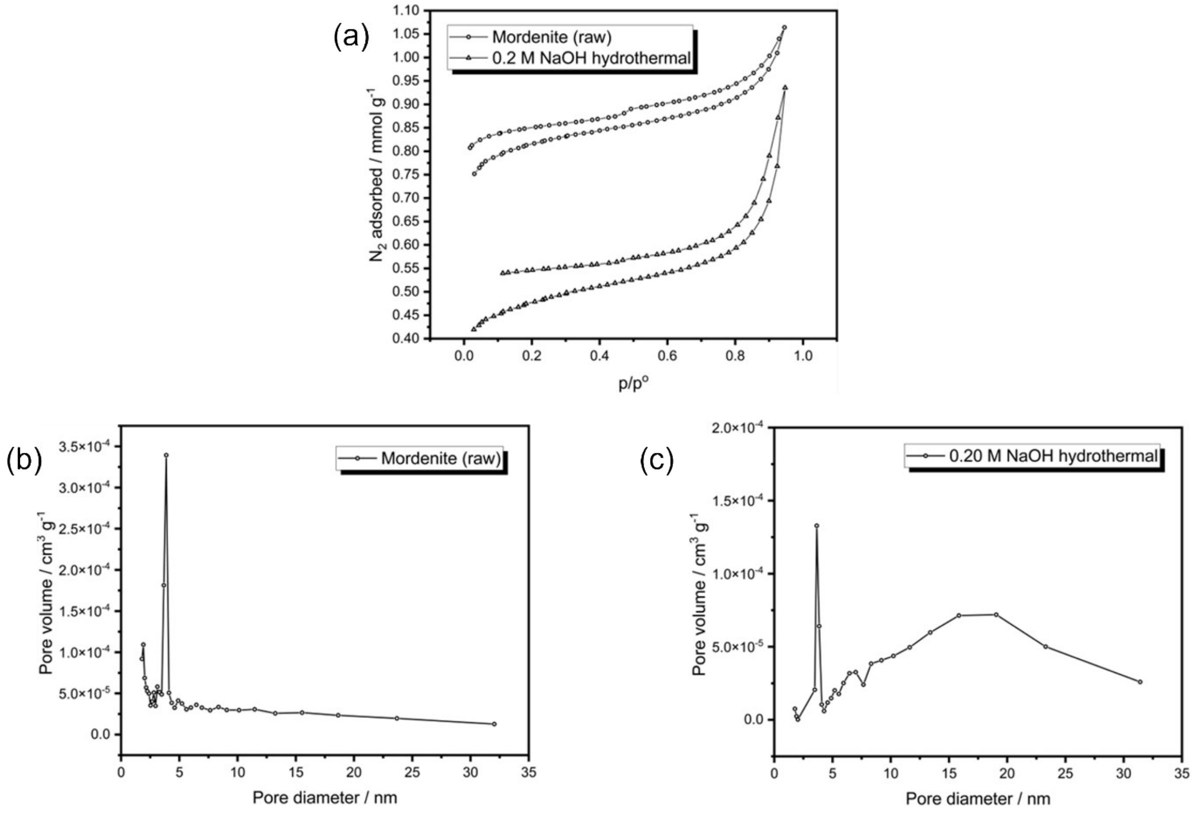

Fig. 15. N_2 adsorption porosimetry data. (a) N_2 adsorption/ desorption isotherms. (b) Pore distribution of mordenite. (c) Pore distribution of 0.20 M NaOH hydrothermally treated mordenite.

Table 3. Surface area of mordenite and 0.20 M NaOH hydrothermally treated mordenite.

Material	BET surface area / $m^2\ g^{-1}$
Mordenite (raw)	72(1)
0.20 M NaOH Hydrothermal	41(1)

6. Peak width fitting

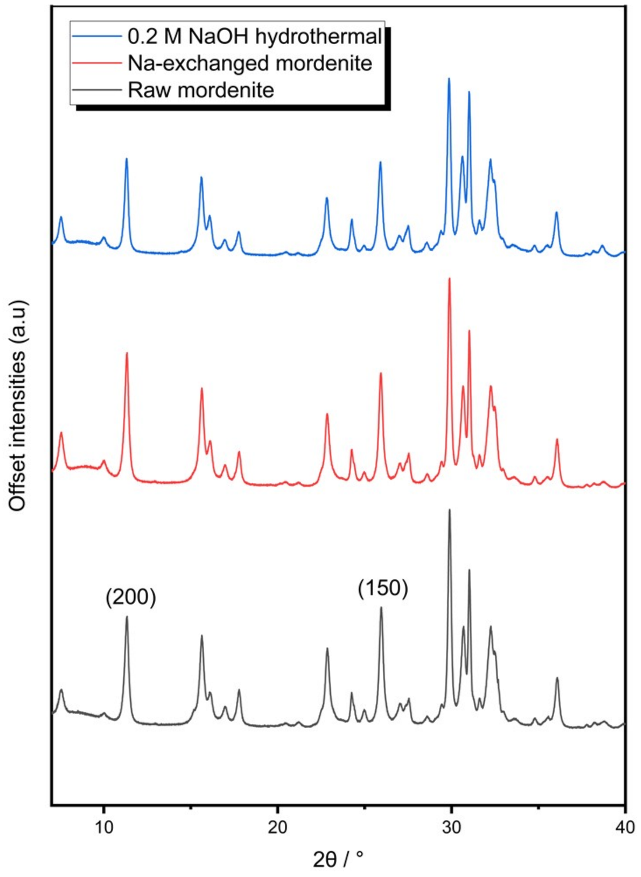

Fig.16 PXRD data for raw mordenite, Na-exchanged mordenite and 0.20M NaOH hydrothermally treated mordenite. (200) and (150) Bragg reflections are labelled on the pattern associated with the raw mordenite (λ = 1.79 Å).

Table 4. Positions and full-width half maximum values for the (200) and (150) reflections in PXRD patterns obtained from raw mordenite, Na-exchanged mordenite and 0.20 M NaOH hydrothermally treated mordenite.

	(200)		(150)	
Material	Position /2θ	FWHM	Position /2θ	FWHM
Raw mordenite	11.31(1)	0.29(3)	25.96(1)	0.28(3)
Na-exchanged mordenite	11.31(1)	0.29(3)	25.93(1)	0.28(3)
0.20 M NaOH	11.30(1)	0.26(3)	25.90(1)	0.29(4)

7. Adsorption studies

Caesium adsorption data

50, 125, 250 and 500 ppm caesium solutions ($CsNO_3$, Fisher, 99.8 %) were allowed to reach equilibrium with 0.018 g zeolite material (Mud Hills clinoptilolite, Na-mordenite, MOR/GIS composite A (0.50 M NaOH treatment concentration, 32:56 MOR: GIS ratio) and 'fully converted' material B (0.70 M NaOH). Uptake (q_e) data as a function of equilibrium concentration is shown in Figure 17. Langmuir, Freundlich and Temkin isotherm models were fitted linearly using regression analysis[5, 6]; Langmuir-Freundlich isotherm models were fitted non-linearly using OriginPro using a Levenberg Marquardt iteration algorithm until convergence was reached (Chi-Sqr tolerance value 1E-9)[6].

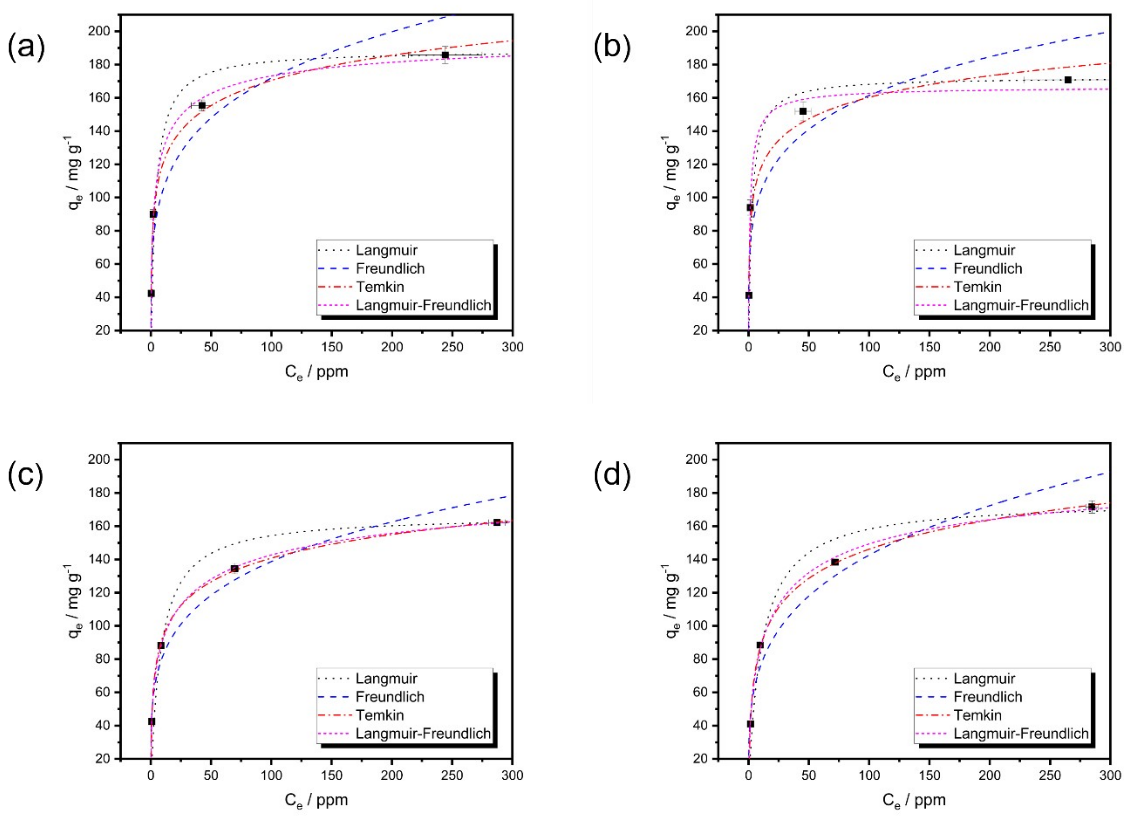

Fig. 17 Caesium equilibrium adsorption isotherms. (q_e) plotted as a function of equilibrium concentration (C_e) for: (a) Mud Hills clinoptilolite, (b) Na-exchanged mordenite, (c) composite material, A (0.50 M NaOH treatment concentration, 32:56 MOR: GIS ratio), (d) 'fully converted' zeolite P, B (0.70 M NaOH solution treatment concentration). Langmuir, Freundlich, Temkin and Langmuir-Freundlich isotherms are plotted.

Calculated parameters from the variety of fitting models are reported in Table 5. Visual inspection suggests that the best fit is obtained using the Langmuir-Freundlich model; maximum capacities (q_{max}) are therefore reported based off these fits.

Table 5. Isotherm parameter summary for Mud Hills clinoptilolite (MH-HEU), Na-exchanged mordenite (Na-MOR), MOR/GIS composite material A (32:56 MOR: GIS ratio) and 'fully converted' zeolite P material B.

	Langmuir			Freundlich			Temkin			Langmuir-Freundlich			
Material	q_m	b	R^2	n	K_f	R^2	B_t	A_t	R^2	q_{max}	K	n	R^2
MH-HEU	189(4)	0.26(19)	0.999	0.21(4)	63(2)	0.927	111(7)	25(3)	0.993	203(23)	0.51(11)	0.53(13)	0.995
Na-MOR	172(3)	0.39(27)	1.000	0.19(6)	65(2)	0.864	129(19)	53(15	0.962	167(10)	0.89(22)	0.81(26)	0.990
A	167(6)	0.12(7)	0.998	0.23(3)	48(2)	0.972	118(2)	9.3(4)	1.000	210(7)	0.28(1)	0.44(2)	1.000
B	175(6)	0.092(44)	0.998	0.27(4)	41(2)	0.952	95(2)	3.1(3)	0.999	207(23)	0.20(3)	0.56(10)	0.997

Strontium adsorption data

20, 50, 125, 250, 400 and 500 ppm strontium solutions ($Sr(NO_3)_2$, Fisher) were allowed to reach equilibrium with 0.018 g zeolite material (Mud Hills clinoptilolite, Na-mordenite, MORGIS composite (A, 0.50 M NaOH treatment concentration, 32:56 MOR: GIS ratio) and 'fully converted' material (B, 0.70 M NaOH)). Uptake (q_e) data as a function of equilibrium concentration is shown in Figure 18. For materials A and B, Langmuir, Freundlich, Temkin isotherm models are fitted linearly using regression analysis[5, 6]; Langmuir-Freundlich isotherm models are also fitted non-linearly using OriginPro using a Levenberg Marquardt iteration algorithm until convergence was reached (Chi-Sqr tolerance value 1E-9)[6]. For Mud Hills clinoptilolite and Na-mordenite, Freundlich, Temkin isotherm models are fitted linearly using regression analysis[5, 6]; Langmuir-Freundlich isotherm models are also fitted non-linearly using OriginPro using a Levenberg Marquardt iteration algorithm until convergence was reached (Chi-Sqr tolerance value 1E-9)[6].

Where appropriate, calculated parameters from the variety of fitting models are reported in Table 6. Visual inspection suggests that the best fit is obtained using the Langmuir-Freundlich model; maximum capacities (q_{max}) are therefore reported based off these fits.

Table 6. Isotherm parameter summary for Mud Hills clinoptilolite (MH-HEU), Na-exchanged mordenite (Na-MOR), MOR/GIS composite material A and 'fully converted' zeolite P material B.

	Langmuir			Freundlich			Temkin			Langmuir-Freundlich			
Material	q_m / mg g^{-1}	b	R^2	n	K_f	R^2	B_t	A_t	R^2	q_{max} / mg g^{-1}	K	n	R^2
MH-HEU				0.11(3)	30(2)	0.887	663(9)	1200(100)	0.9085	56(5)	2.1(10)	0.42(13)	0.963
Na-MOR				0.09(3)	25(2)	0.961	890(10)	2200(200)	0.948	54(17)	1.0(8)	0.23(11)	0.923
A	93(2)	0.090(5)	0.999	0.27(3)	23(2)	0.987	213(5)	8(1)	0.979	130(22)	0.20(4)	0.42(8)	0.994
B	125(5)	0.15(8)	0.993	0.24(3)	35(2)	0.964	176(5)	25(2)	0.984	146(15)	0.38(7)	0.42(6)	0.997

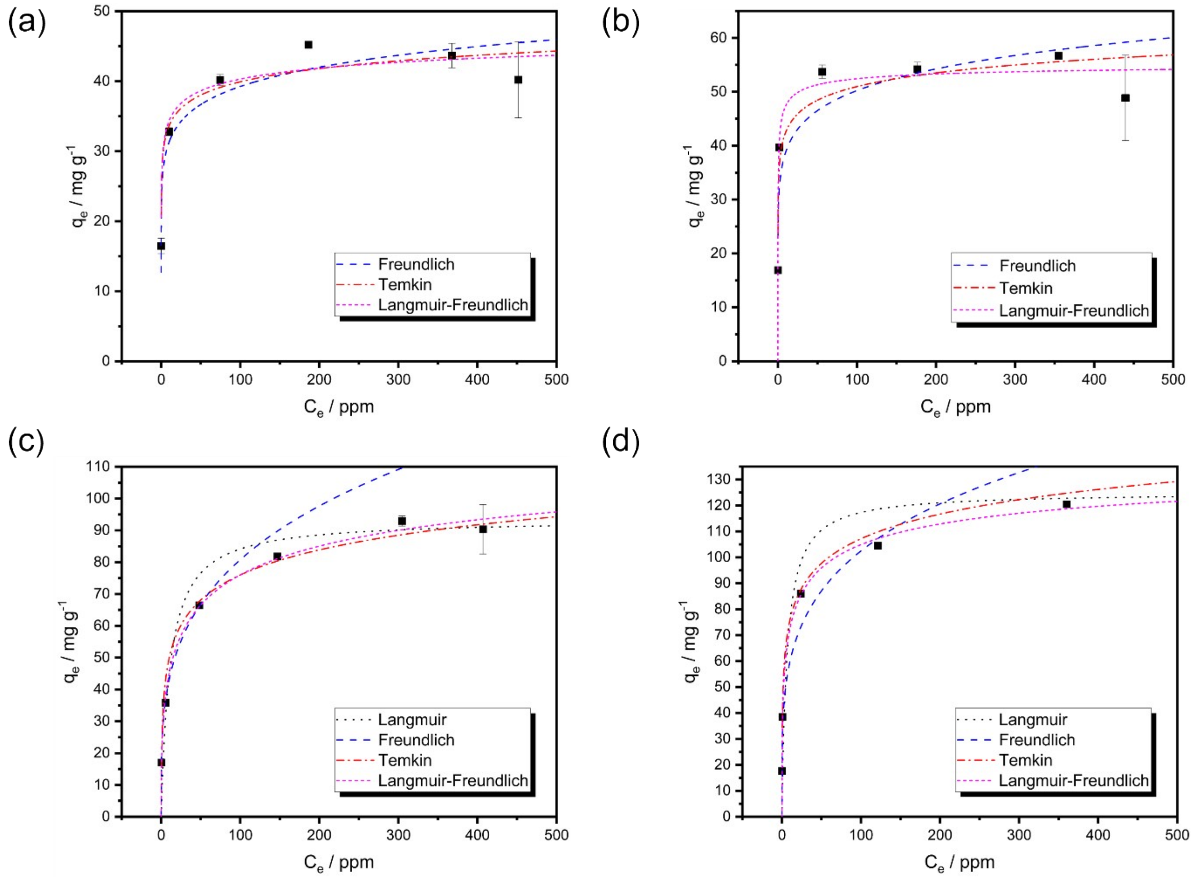

Fig. 18 Caesium equilibrium adsorption isotherms. (q_e) plotted as a function of equilibrium concentration (C_e) for: (a) Mud Hills clinoptilolite, (b) Na-exchanged mordenite, (c) composite material, A (0.50 M NaOH treatment concentration), (d) 'fully converted' zeolite P, B (0.70 M NaOH solution treatment concentration). Langmuir, Freundlich, Temkin and Langmuir-Freundlich isotherms are plotted where appropriate.

8. Non industrially deployed ion-exchange materials

Cs exchange materials

Table 7. Non industrially deployed ion-exchange materials for caesium adsorption.

Material	Cs capacity mg g^{-1}	References
MIL-101-SO3H (MOF)	453	[7]
Struvite	600-700[8]	[8, 9]
Illite (clay)	-	[10]
KTSS	450	[11]
Umbite	-	[12, 13]

Sr exchange materials

Table 8. Non industrially deployed ion-exchange materials for strontium adsorption.

Material	Sr capacity mg g^{-1}	References
Antimony silicates	65-75	[14]
Apatite	8-10	[15]
Mesoporous manganese oxides	200-220	[16]

9. Granular PXRD patterns

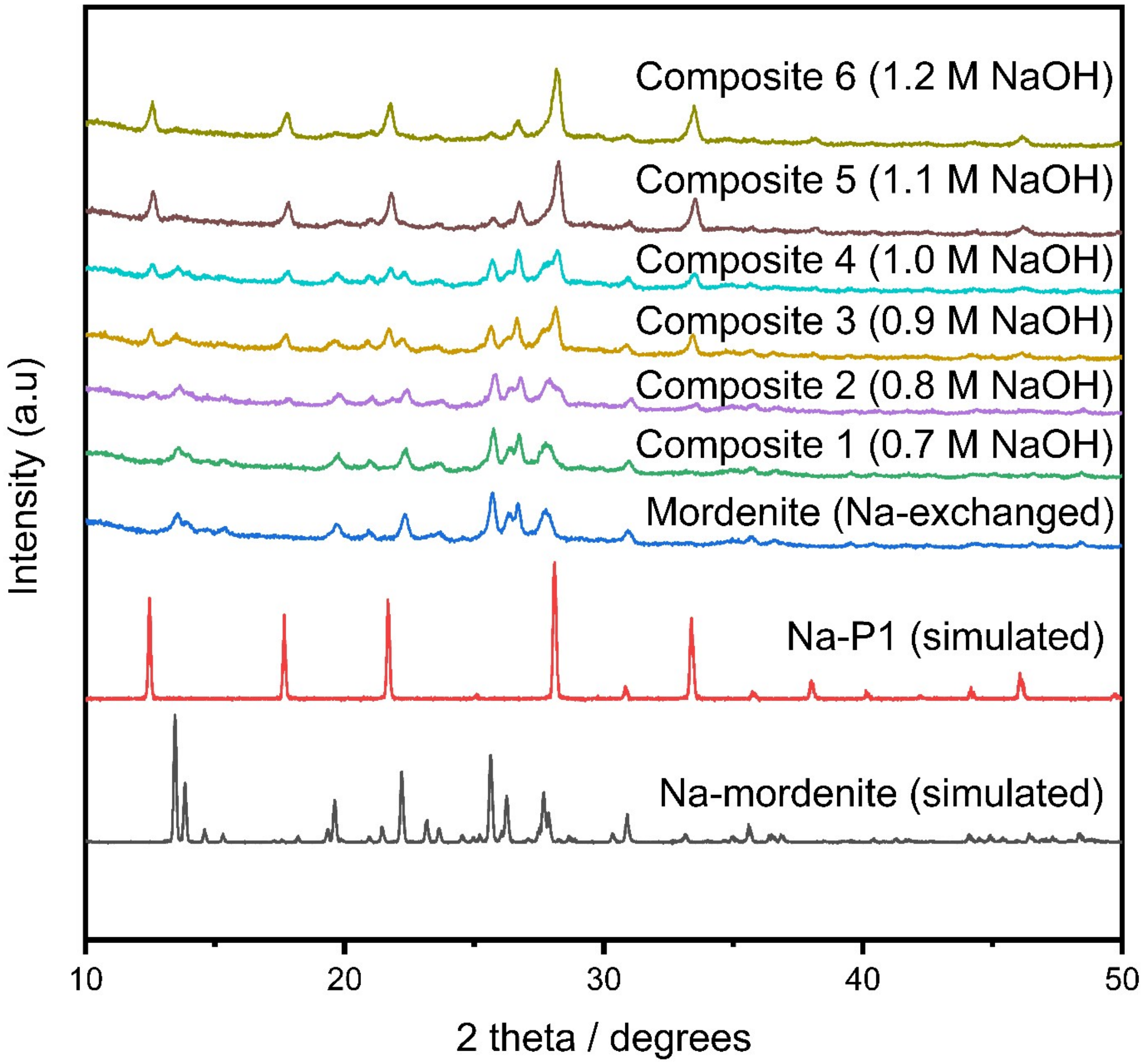

Fig. 19 PXRD patterns of granular composite materials. Simulated patterns for Na-mordenite and Na-P1 are also displayed (λ = 1.5406 Å).

10. Rietveld refinements of granular zeolite composites

Refinements were completed using GSAS ii[3] assuming the sole presence of three phases: Na-MOR, Na-P1 and quartz. A summary of fit qualities and weight fractions is shown in Table 9.

Mordenite

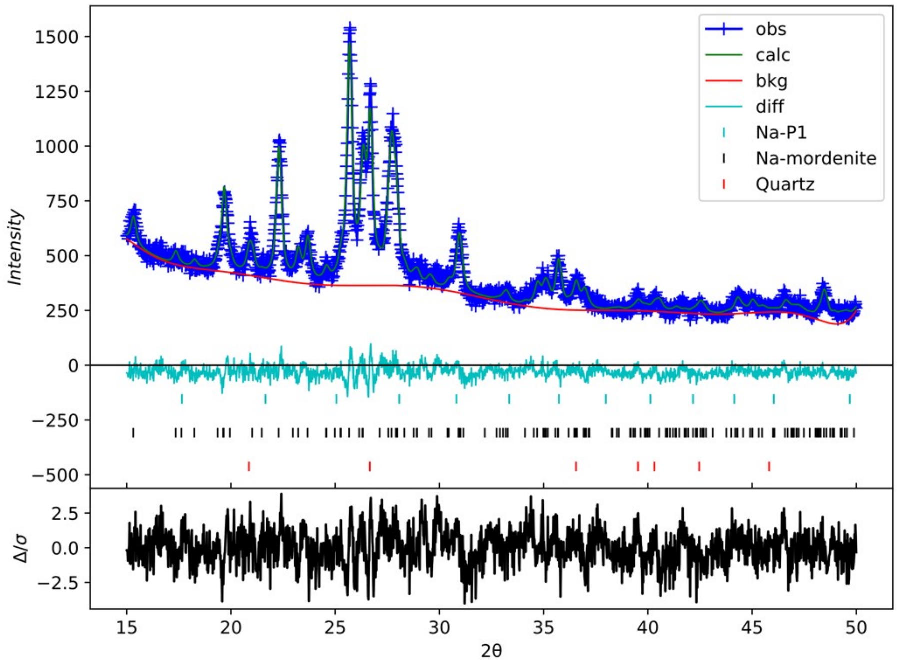

Fig. 20 Rietveld refinement of PXRD data for granular mordenite material (Na-exchanged).

Composite 1 (0.7 M NaOH)

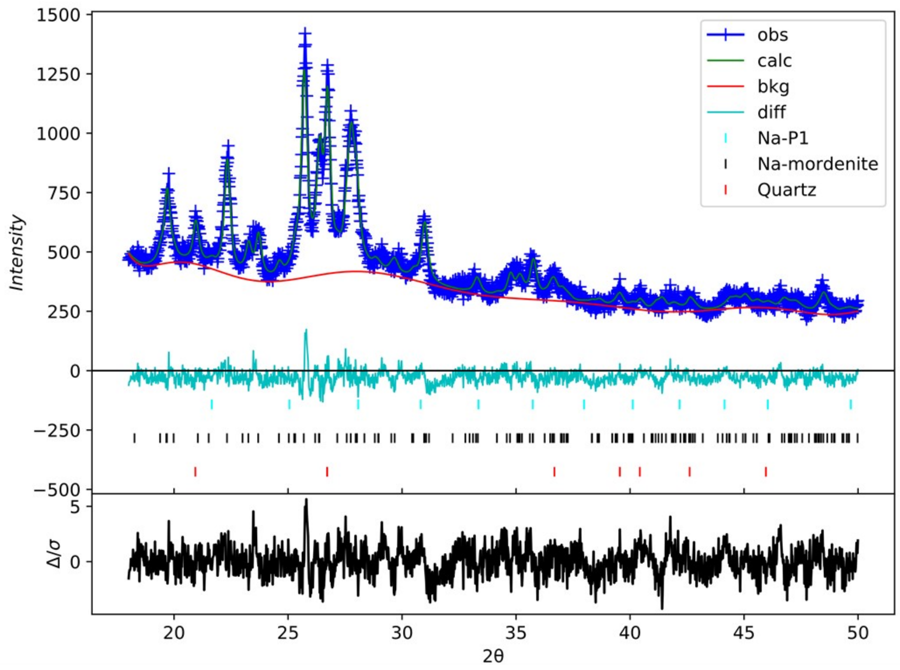

Fig. 21 Rietveld refinement of PXRD data for 0.7 M NaOH treated granular mordenite.

Composite 2 (0.8 M NaOH)

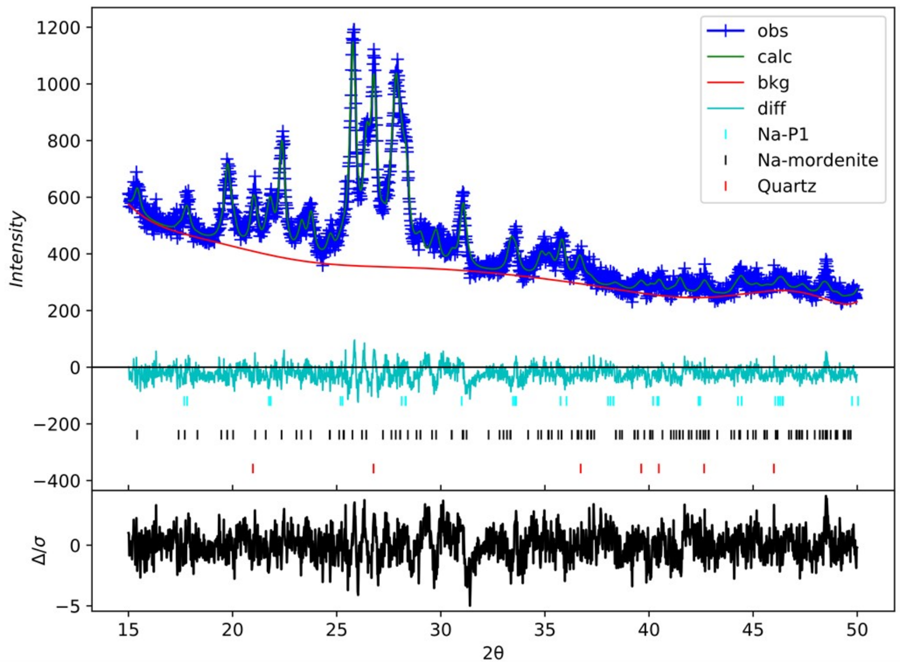

Fig. 22 Rietveld refinement of PXRD data for 0.8 M NaOH treated granular mordenite.

Composite 3 (0.9 M NaOH)

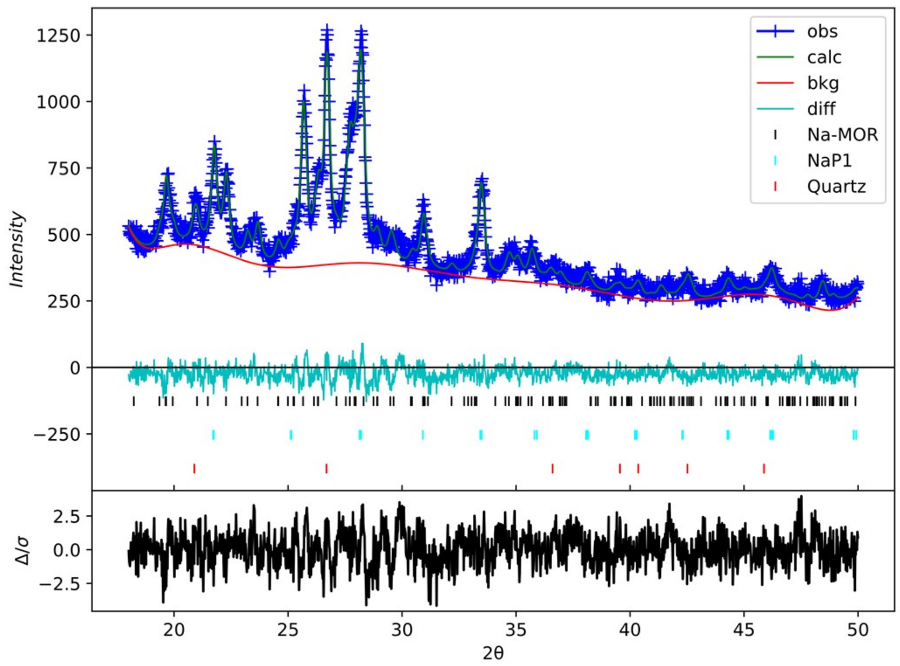

Fig. 23 Rietveld refinement of PXRD data for 0.9 M NaOH treated granular mordenite.

Composite 4 (1.0 M NaOH)

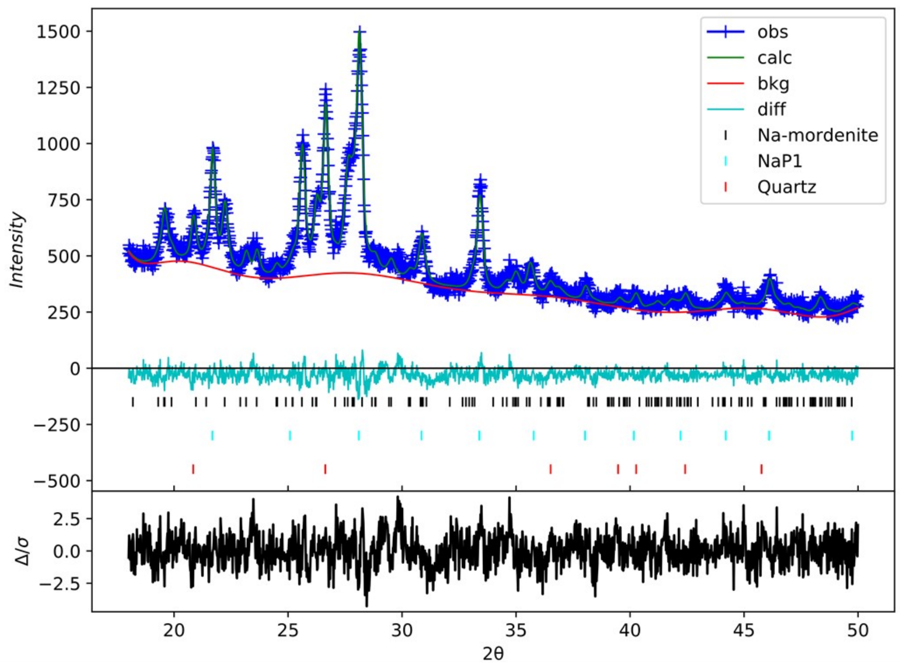

Fig. 24 Rietveld refinement of PXRD data for 1.0 M NaOH treated granular mordenite.

Composite 5 (1.1 M NaOH)

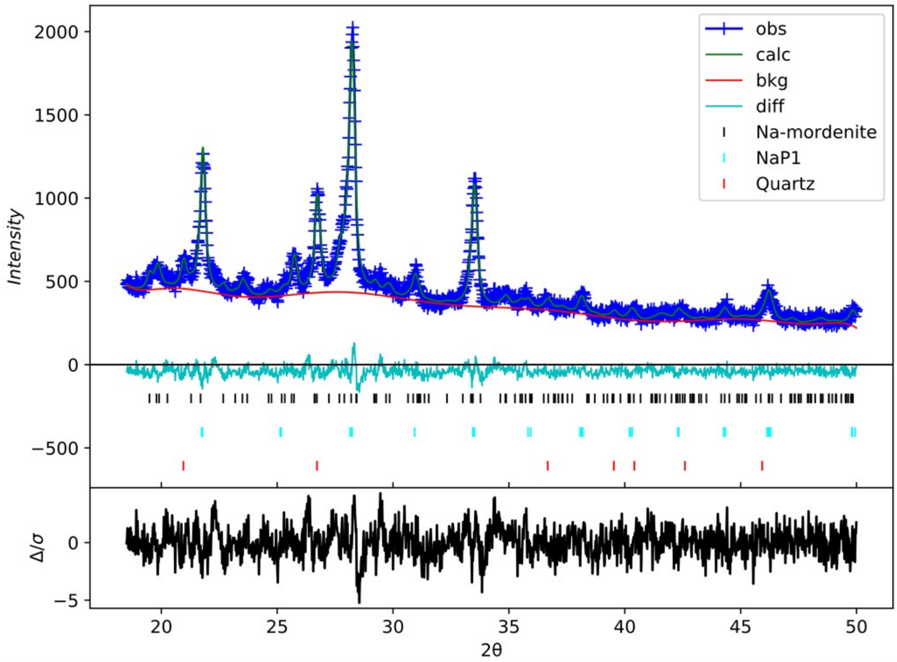

Fig. 25 Rietveld refinement of PXRD data for 1.1 M NaOH treated granular mordenite.

Composite 6 (1.2 M NaOH)

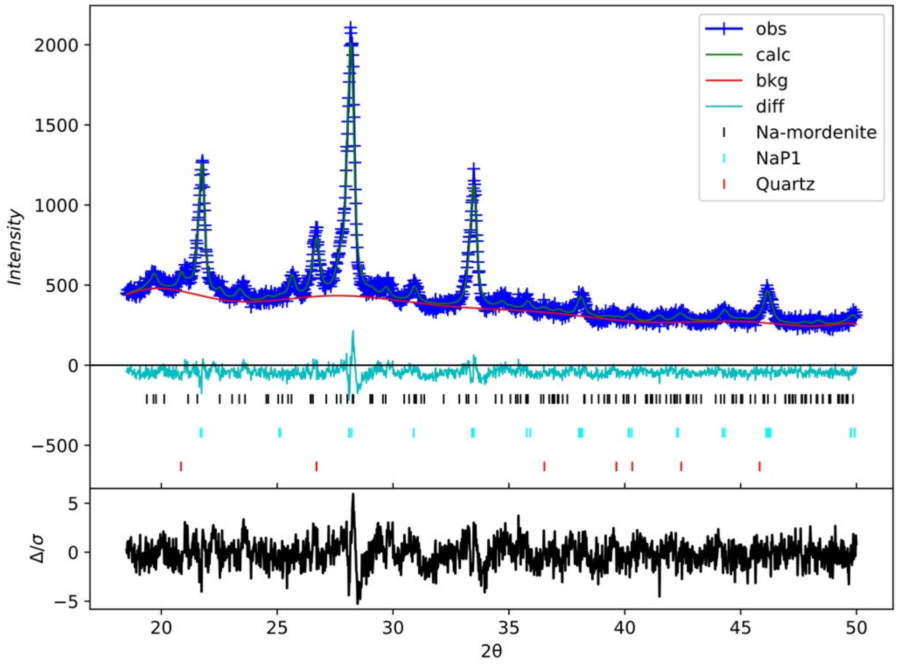

Fig. 26 Rietveld refinement of PXRD data for 1.2 M NaOH treated granular mordenite.

Table 9: Summary of refinement results of granular materials.

Material	wR / %	GOF	MOR W_f	GIS W_f	Quartz W_f
**Mordenite**	6.12	1.25	0.92(1)	0.00(1)	0.08(1)
**Composite 1**	6.30	1.32	0.91(1)	0.01(1)	0.08(1)
**Composite 2**	5.89	1.23	0.85(1)	0.09(1)	0.06(1)
**Composite 3**	5.89	1.23	0.62(1)	0.28(1)	0.10(1)
**Composite 4**	5.64	1.19	0.57(1)	0.34(1)	0.09(1)
**Composite 5**	5.99	1.26	0.25(1)	0.67(1)	0.08(1)
**Composite 6**	6.22	1.31	0.22(6)	0.73(8)	0.05(2)

11. Rietveld refinements of local diffraction patterns of granules

Refinements were completed using GSAS ii[3] assuming the sole presence of three phases: Na-MOR, Na-P1 and quartz (excluding mordenite which assumed only the presence Na-MOR and quartz).

Point P (centre of mordenite granule)

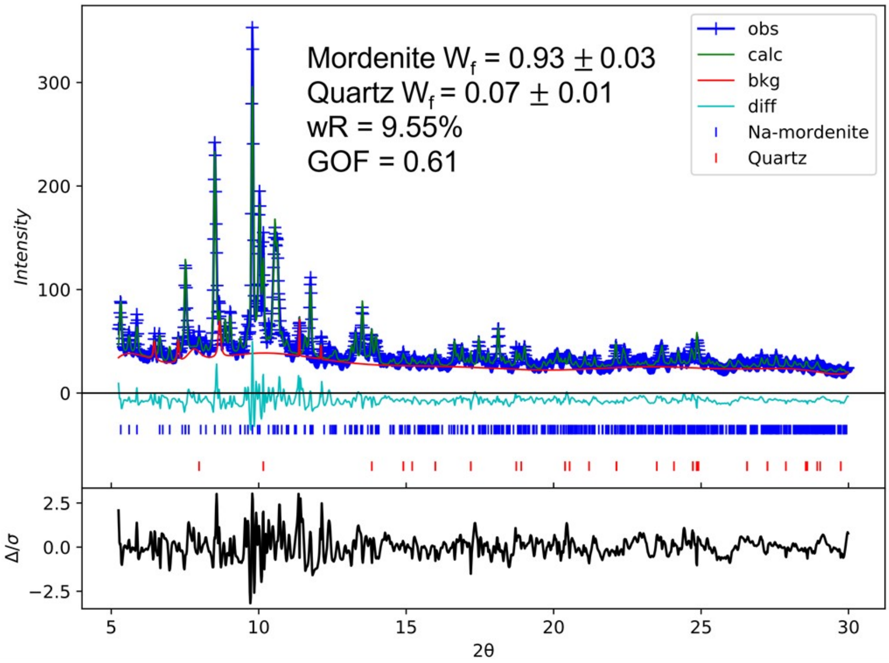

Fig. 27 Rietveld refinement of PXRD data for point P including estimated weight fractions. Some unidentified peaks have been incorporated into the background.

Point M (centre of composite 4 granule)

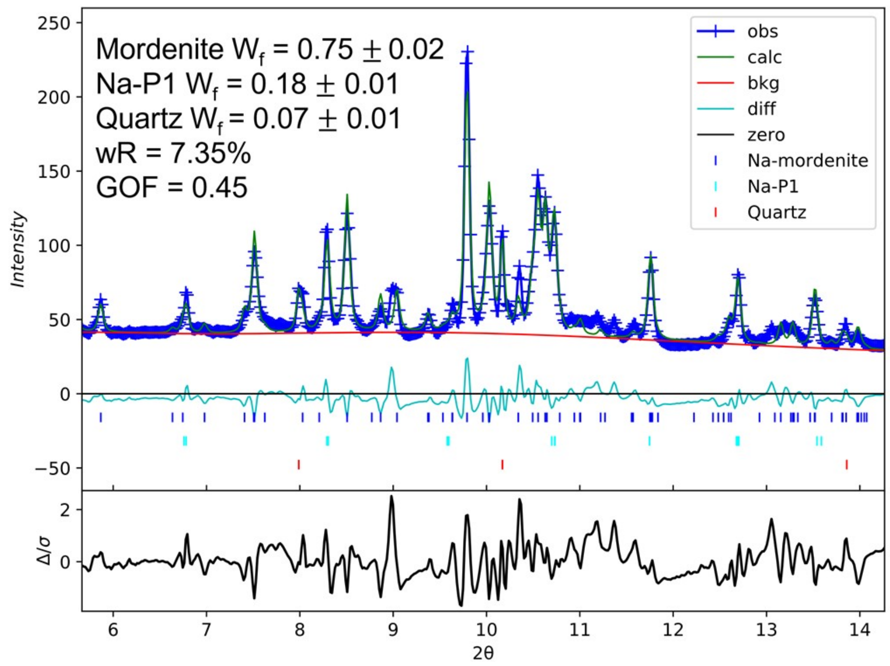

Fig. 28 Rietveld refinement of PXRD data for point M including estimated weight fractions.

Point N (outer shell of composite 4 granule)

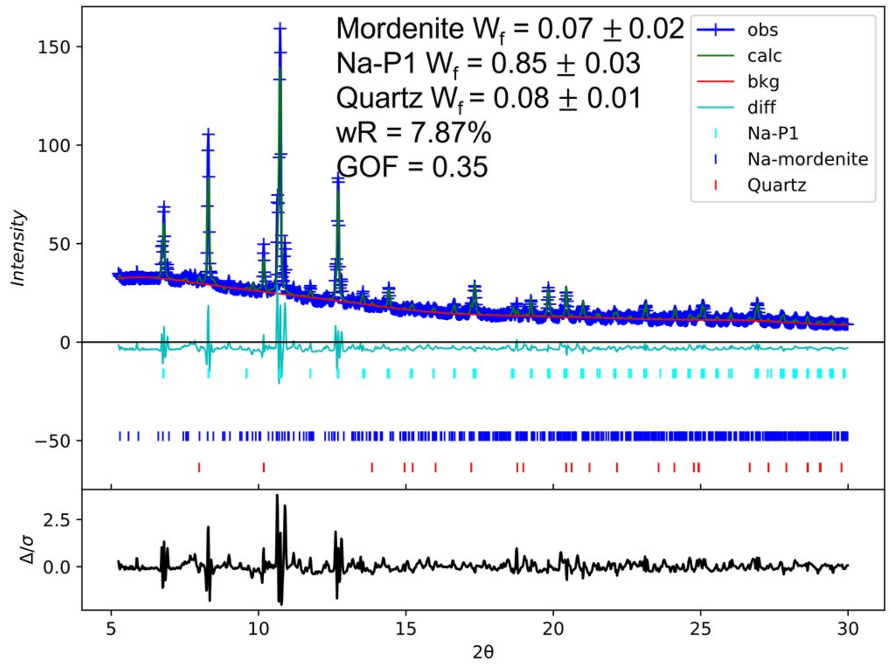

Fig. 29 Rietveld refinement of PXRD data for point N including estimated weight fractions.

Point R (centre of composite 5 granule)

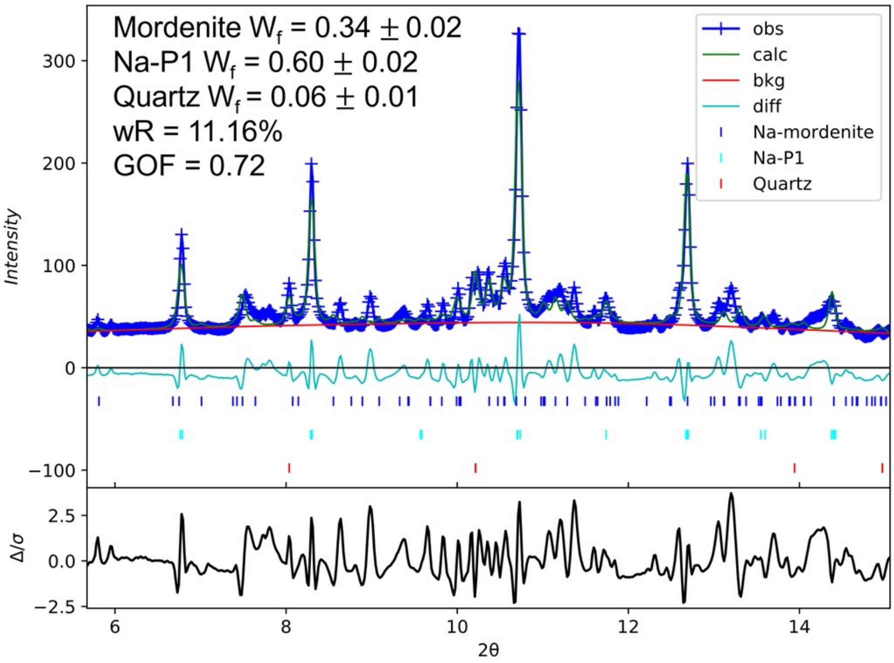

Fig. 30 Rietveld refinement of PXRD data for point R including estimated weight fractions.

12. In-situ image-guided diffraction studies of ion-exchange

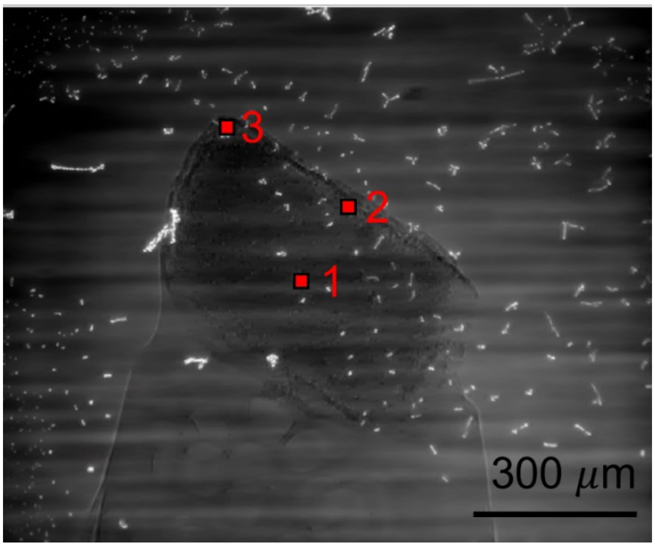

Fig. 31 Diffraction beam trajectories used during in-situ image-guided diffraction studies of ion-exchange.

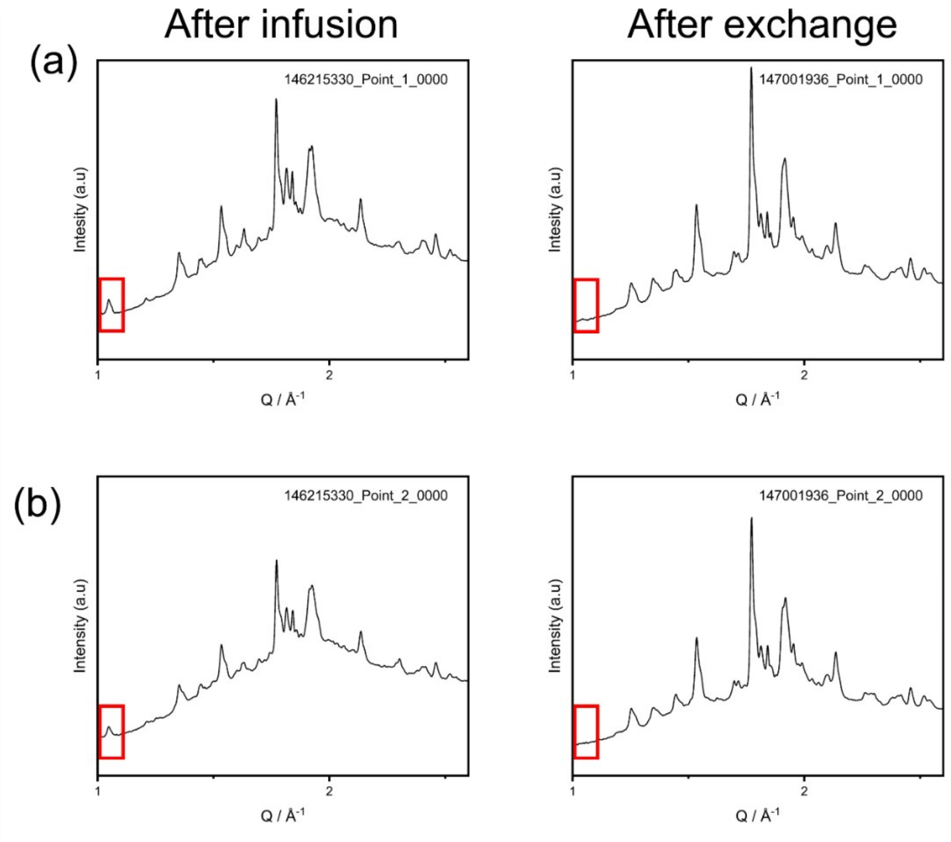

Fig. 32 PXRD patterns obtained from Point 1 and Point 2 diffraction beam trajectories. MOR (130) reflection is highlighted before and after exchange. (a) Point 1. (b) Point 2.

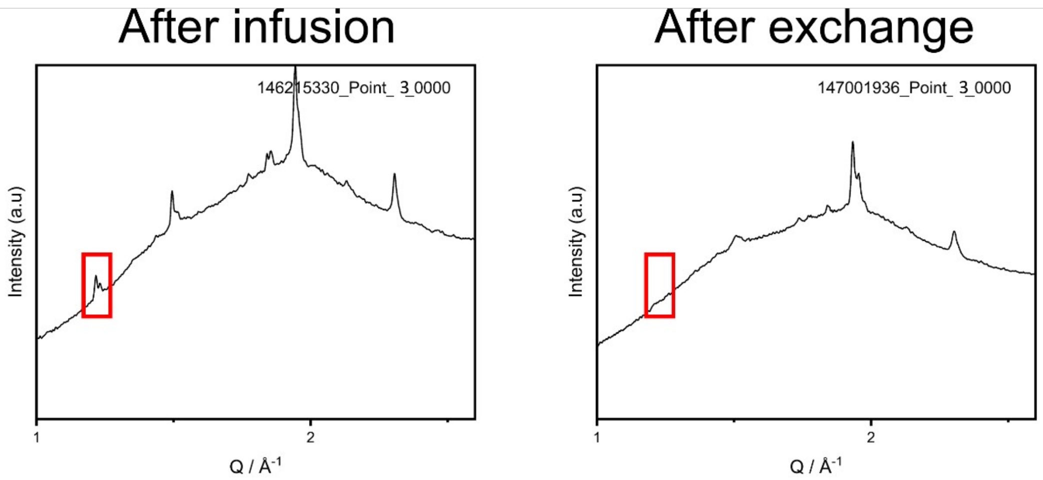

Fig. 33 PXRD patterns obtained from Point 3 diffraction beam trajectory. GIS (200) reflection is highlighted before and after exchange.

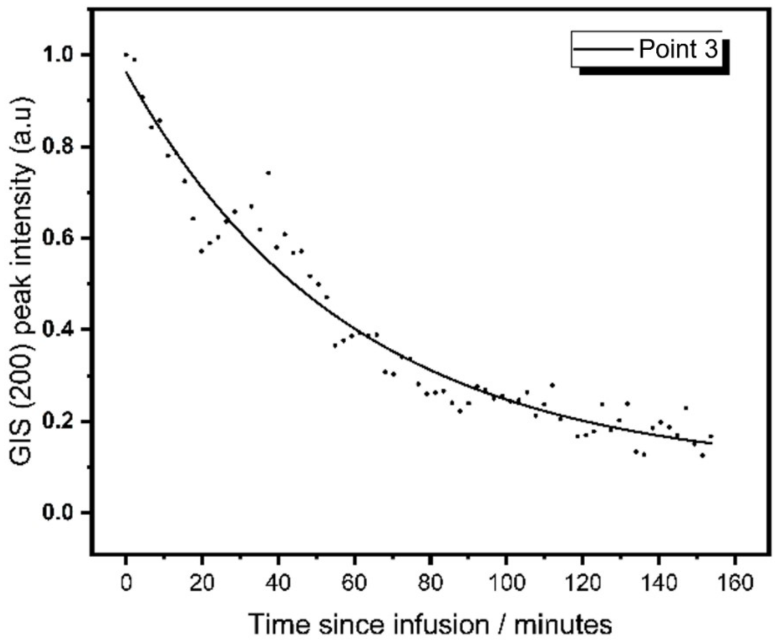

Fig. 34 GIS (200) reflection intensity as a function of time during in-situ ion-exchange experiment.

13. Rapid ion-exchange uptake curve fitting and derivation

A two-parameter exponential decay (Eq. 1) function was fitted to the data points using a Levenberg Narquardt iteration algorithm until convergence was reached (Chi-Sqr tolerance value 1E-9). This curve then represents all data for a single material. A two-parameter exponential decay function was selected due to excellent fits with experimental data points.

(Eq. 1): $$y = y_0 + A_1 e^{-x/t_1} + A_2 e^{-x/t_2}$$

Table 10. Exponential parameters and R^2 for fits of RIX data to two-parameter exponential decay functions. The concentration of NaOH used to form the composites is shown in brackets for Composites 2 and 4.

Material	Radionuclide	Y_0	A_1	T_1	A_2	T_2	R^2
**Mud Hills clinoptilolite**	Cs-137	2.18	22.2	55.7	75.7	349	0.993
	Sr-90	-0.84	27.8	51.9	72.3	662	0.987
**Mordenite**	Cs-137	3.14	32.4	58.0	64.4	366	0.997
	Sr-90	-16.7	32.9	35.3	83.7	1399	0.980
**Composite 2 (0.8M NaOH)**	Cs-137	3.46	48.3	72.1	48.2	429	0.995
	Sr-90	3.39	33.0	32.8	63.6	598	0.991
**Composite 4 (1.0 M NaOH)**	Cs-137	4.43	46.3	53.5	49.2	412	0.999
	Sr-90	4.38	44.8	33.3	50.8	650	0.997

Approximation of sorption probability in rapid ion-exchange experiments

For every volume of liquor being extracted from the reservoir in a given time, an equal volume is extruded back into the reservoir at a new concentration. These concentrations can be linked by Eq. 2, where α is the probability of sorption.

(Eq. 2): $$Column\ output\ volume = Column\ input\ volume \times \alpha$$

Considering this scenario, where a given volume is flown though the column (V_f) in a set time, the following equation (Eq. 3) can be constructed, where C_x and C_{x-1} are the final and initial concentration of the reservoir, which has associated volume (V_r):

(Eq. 3): $$C_x = \frac{(V_r - V_f)C_{x-1}}{V_r} + \frac{V_f(1-\alpha)C_{x-1}}{V_r}$$

Considering the change in reservoir concentrations in a given time, $\frac{dC}{dt}$:

$$\frac{dC}{dt} = C_x - C_{x-1}$$

Using Eq. 3:

$$\frac{dC}{dt} = C_{x-1}(V_r - V_f\alpha - 1)$$

$$\int \frac{1}{C} dc = \int V_r - V\alpha - 1\ dt$$

$$C = 100e^{\frac{-\alpha V_f t}{V_r}}$$

This model does not account for the initial volume of water in the tubing or the aliquots removed for analysis. Further, the initial concentration (t=0) is given the arbitrary value of 100 for easy comparison to percentage uptake curve. In reality, the probability of sorption, α, decreases as reservoir concentration decreases predominantly due to the reducing concentration of the column input liquor. Therefore, we would not expect this model to fit any of our uptake curves; it is to be used only as a tool to estimate α for different materials at different points of the experiment. A comparison of this model (using three sorption probabilities: α= 1, 0.5, 0.25) to Composite 4 is displayed (Fig. 36).

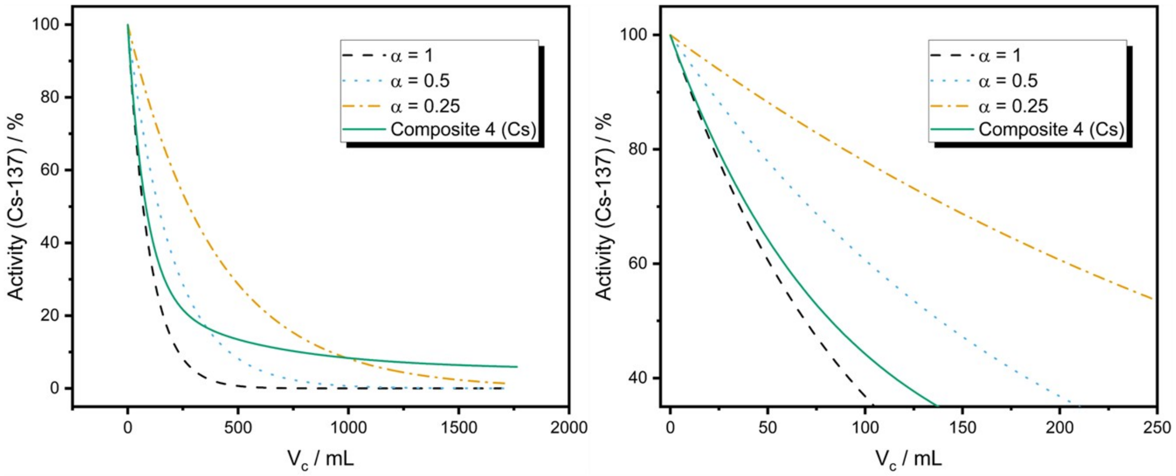

Fig. 35 Comparison of different probabilities of sorption (α= 1, 0.5, 0.25) for the model $C = 100e^{\frac{-\alpha V_f t}{V_r}}$**.** Cs-137 data for Composite 4 is also displayed.

14. xCT of Mud Hills clinoptilolite

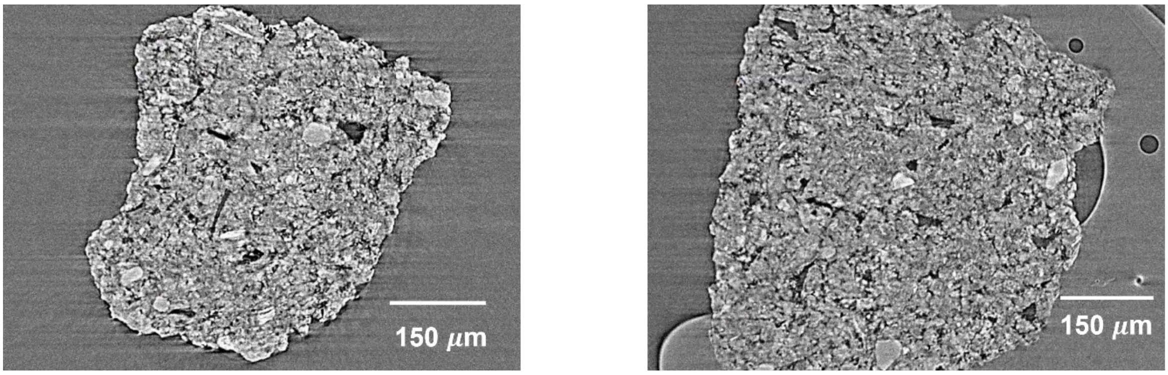

Figure 36. xCT cross-sections of Mud Hills clinoptilolite.

15. Batch rate studies

Caesium rate studies

250 ppm caesium solutions were made up from $CsNO_3$ (Fisher, 99.8%). 0.30 g zeolite material (Mud Hills clinoptilolite, Na-mordenite, MORGIS composite A (0.50 M NaOH treatment concentration, 32:56 MOR: GIS ratio) and 'fully converted' material B (0.70 M NaOH)) was added to 250 mL stirring solution before 1 mL aliquots were extracted at set time intervals (0, 2, 5, 15, 60, 180, 360 and 1440 minutes). Aliquots were passed through a 0.22 μm filter prior to ten-fold dilution in 2% nitric acid and analysis by ICP-MS. The 2nd order Kinetic models was fitted to a plot of time versus uptake (q_t) (Fig. 38).

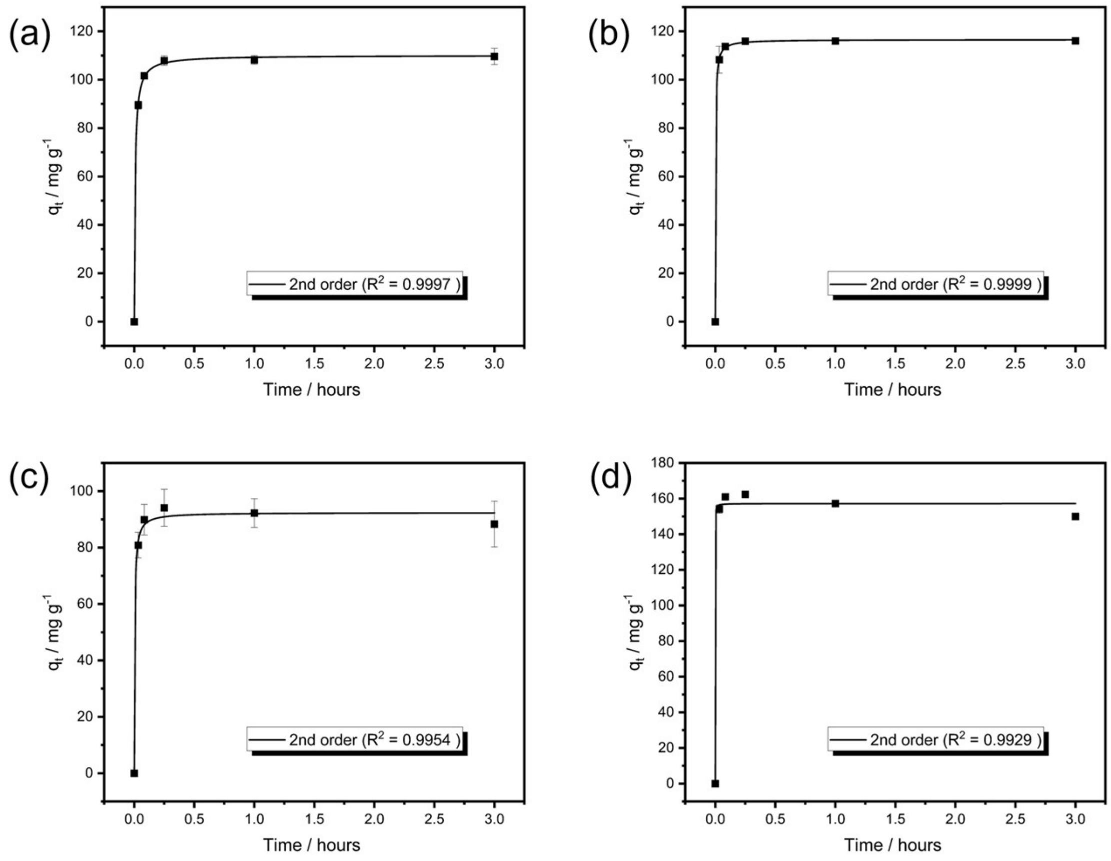

Fig. 37: Caesium kinetic isotherms for: (a) Mud Hills clinoptilolite, (b) Na-exchanged mordenite, (c) composite material, A (0.50 M NaOH treatment concentration), (d) 'fully converted' zeolite P, B (0.70 M NaOH solution treatment concentration). 2nd order rate equation plots are also shown.

The 2nd order kinetic model provided excellent fits ($R^2 > 0.99$) for all 4 materials (Table 11). Equilibrium was very quickly reached across the samples (< 15 mins). Rate constants appear to increase for treated materials; this suggests that transformation has a positive effect on the rate of exchange.

Table 11. 2nd order fitting parameters for caesium kinetic isotherms.

Material	q_e	k	R^2
Mud Hills clinoptilolite	110(1)	1.22(8)	0.9997
Na mordenite	117(1)	3.49(26)	0.9999
A	92(2)	2.61(97)	0.9954
B	157(3)	40(330)	0.9929

Strontium rate studies

250 ppm strontium solutions made up from $Sr(NO_3)_2$ (Fisher). 0.30 g zeolite material (Mud Hills clinoptilolite, Na-mordenite, MORGIS composite A (0.50 M NaOH treatment concentration, 32:56 MOR: GIS ratio) and 'fully converted' material B (0.70 M NaOH)) was added to 250 mL stirring solution before 1 mL aliquots were extracted at set time intervals (0, 2, 5, 15, 60, 180, 360 and 1440 minutes). Aliquots are passed through a 0.22 μm filter prior to ten-fold dilution in 2% nitric acid and analysis by ICP-OES. 2nd order kinetic / Elovich models were fitted to a plot of time versus uptake (q_t) (Fig. 39).

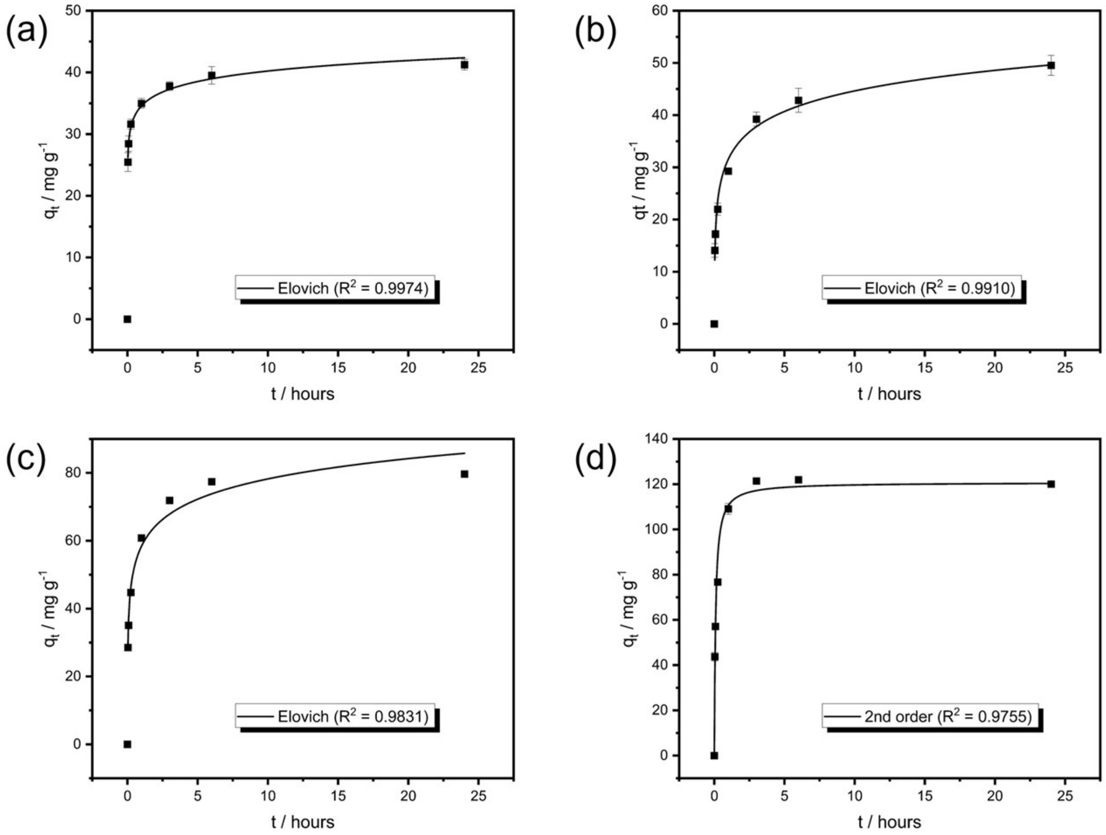

Fig. 38: Strontium kinetic isotherms for: (a) Mud Hills clinoptilolite, (b) Na-exchanged mordenite, (c) composite material, A (0.50 M NaOH treatment concentration), (d) 'fully converted' zeolite P, B (0.70 M NaOH solution treatment concentration). Elovich rate equation plots are also shown for Mud Hills clinoptilolite, Na mordenite and composite B; a 2nd order rate equation is shown for 'fully converted' material B.

The rate of strontium uptake was significantly slower than that for caesium; only the 'fully converted' material B reached equilibrium within the 24-hour experimental timescale. The second order kinetic model fits were inferior compared to analogous caesium data (Table 12), although material B's data was fitted well (R^2 = 0.9755). In order to better model the data, the Elovich model was also fitted to the datasets (Table 13).

Table 12. 2nd order fitting parameters for strontium kinetic isotherms.

Material	q_e	k	R^2
Mud Hills clinoptilolite	43(4)	0.12(6)	0.8926
Na mordenite	38(2)	1.23(35)	0.9633
A	75(4)	0.13(5)	0.9429
B	121(4)	0.09(2)	0.9755

The Elovich model provided improved fits for Mud Hills clinoptilolite, Na mordenite and Composite material A ($R^2 > 0.98$) (Table 13). The fit to data for the 'fully converted' material B was inferior to the 2nd order kinetic model.

Table 13. Elovich fitting parameters for strontium kinetic isotherms.

Material	α	β	R^2
Mud Hills clinoptilolite	1471(345)	0.18(1)	0.9910
Na mordenite	3211998(1996703)	0.41(2)	0.9974
A	7490(3260)	0.12(1)	0.9831
B	17878(16217)	0.08(2)	0.9421

In order to compare rates of uptake between models, the estimated time to reach 95% equilibrium has been calculated (Table 14).

Table 14. Estimated time to 95% equilibrium loading determined from kinetic models.

Material	Model	Time to 95% q_e / hours
Mud Hills clinoptilolite	Elovich	60(15)
Na mordenite	Elovich	20(12)
A	Elovich	15(6)
B	2nd order kinetic	2(1)

From this data it is apparent that the hydrothermal transformations have a positive effect on the rate of strontium uptake, especially when the drastically enhanced strontium capacity is considered (SI. 7).

16. References

1. W. van Aarle, W. J. Palenstijn, J. Cant, E. Janssens, F. Bleichrodt, A. Dabravolski, J. De Beenhouwer, K. J. Batenburg and J. Sijbers, *Optics Express*, 2016, **24**, 25129-25147.
2. N. T. Vo, M. Drakopoulos, R. C. Atwood and C. Reinhard, *Optics Express*, 2014, **22**, 19078-19086.
3. B. H. Toby and R. B. Von Dreele, *Journal of Applied Crystallography*, 2013, **46**, 544-549.
4. J. Rouquerol, D. Avnir, D. H. Everett, C. Fairbridge, M. Haynes, N. Pernicone, J. D. F. Ramsay, K. S. W. Sing and K. K. Unger, *GUIDELINES FOR THE CHARACTERIZATION OF POROUS SOLIDS*, 1994.
5. M. A. Al-Ghouti and D. A. Da'ana, *J. Hazard. Mater.*, 2020, **393**, 122383.
6. S. Azizian, *Adsorption: Fundamental Processes and Applications (Chapter 6)*, Academic Press, 2021.
7. B. Aguila, D. Banerjee, Z. M. Nie, Y. Shin, S. Q. Ma and P. K. Thallapally, *Chemical Communications*, 2016, **52**, 5940-5942.
8. Z. Q. Li, C. Y. Yang and K. K. Cho, *J. Hazard. Mater.*, 2023, **453**, 131385.
9. Z. W. Lei, X. W. Li, P. W. Huang, H. M. Hu, Z. Li and Q. W. Zhang, *Applied Clay Science*, 2019, **168**, 223-229.
10. C. B. Durrant, J. D. Begg, A. B. Kersting and M. Zavarin, *Science of the Total Environment*, 2018, **610**, 511-520.
11. Z. Z. Jiang, G. L. Liu, C. Ma, Y. F. Guo, J. Duo, M. L. Li and T. L. Deng, *Chemosphere*, 2022, **305**.
12. R. George and J. A. Hriljac, *Mrs Advances*, 2017, **2**, 729-734.
13. A. Nearchou, T. Y. Chen, D. S. Parsons, L. O'Brien, N. A. Hodge, L. Abrahamsen-Mills, P. K. Allan and J. A. Hriljac, *Apl Materials*, 2023, **11**, 011105.
14. K. G. Varshney, U. Sharma, S. Rani and A. Premadas, *Separation Science and Technology*, 1983, **17**, 1527-1543.
15. S. Handley-Sidhu, T. K. Mullan, Q. Grail, M. Albadarneh, T. Ohnuki and L. E. Macaskie, *Scientific Reports*, 2016, **6**, 23361.
16. A.I. Ivanets, V.G. Prozorovich, T.F. Kouznetsova, A.V. Radkevich and A.M. Zarubo, *nvironmental Nanotechnology, Monitoring & Management,*, 2016, **6**, 261-269.
